# Identification of Exciton Complexes in Charge-Tunable Janus W_Se_^S^ Monolayers

**DOI:** 10.1021/acsnano.2c10697

**Published:** 2023-04-14

**Authors:** Matthew S. G. Feuer, Alejandro R.-P. Montblanch, Mohammed Y. Sayyad, Carola M. Purser, Ying Qin, Evgeny M. Alexeev, Alisson R. Cadore, Barbara L. T. Rosa, James Kerfoot, Elaheh Mostaani, Radosław Kalȩba, Pranvera Kolari, Jan Kopaczek, Kenji Watanabe, Takashi Taniguchi, Andrea C. Ferrari, Dhiren M. Kara, Sefaattin Tongay, Mete Atatüre

**Affiliations:** † Cavendish Laboratory, University of Cambridge, 19 J. J. Thomson Avenue, Cambridge, CB3 0HE, U.K.; ‡ Materials Science and Engineering, School for Engineering of Matter, Transport and Energy, Arizona State University, Tempe, Arizona 85287, United States; § Cambridge Graphene Centre, University of Cambridge, 9 J. J. Thomson Avenue, Cambridge, CB3 0FA, U.K.; ∥ Research Center for Functional Materials, National Institute for Materials Science, 1-1 Namiki, Tsukuba 305-0044, Japan; ⊥ International Center for Materials Nanoarchitectonics, National Institute for Materials Science, 1-1 Namiki, Tsukuba 305-0044, Japan

**Keywords:** Janus transition-metal dichalcogenides, W_Se_^S^ monolayers, 2D materials, layered materials, charge tunable, excitons

## Abstract

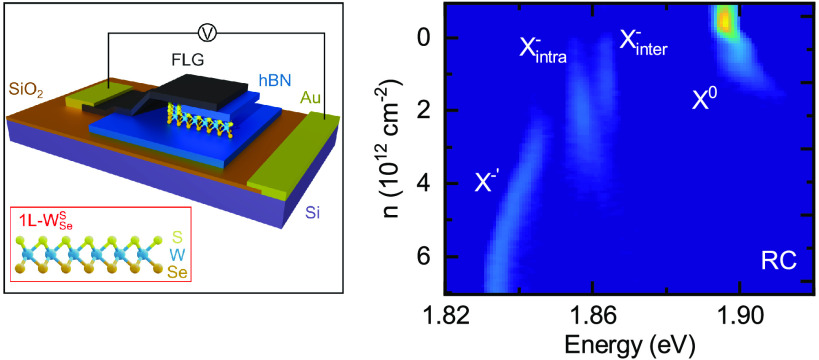

Janus transition-metal dichalcogenide monolayers are artificial materials, where one plane of chalcogen atoms is replaced by chalcogen atoms of a different type. Theory predicts an in-built out-of-plane electric field, giving rise to long-lived, dipolar excitons, while preserving direct-bandgap optical transitions in a uniform potential landscape. Previous Janus studies had broad photoluminescence (>18 meV) spectra obfuscating their specific excitonic origin. Here, we identify the neutral and the negatively charged inter- and intravalley exciton transitions in Janus W_Se_^S^ monolayers with ∼6 meV optical line widths. We integrate Janus monolayers into vertical heterostructures, allowing doping control. Magneto-optic measurements indicate that monolayer W_Se_^S^ has a direct bandgap at the K points. Our results pave the way for applications such as nanoscale sensing, which relies on resolving excitonic energy shifts, and the development of Janus-based optoelectronic devices, which requires charge-state control and integration into vertical heterostructures.

Layered materials are solids with strong intralayer bonds but only weak van der Waals coupling between layers.^1^ These materials have a range of electronic,^2^ optical,^3,7,12^ and topological^4^ properties and can be combined in vertical heterostructures with pristine atomic interfaces, despite mismatched lattice parameters.^5−8^ Direct-bandgap semiconducting transition-metal dichalcogenide (TMD) monolayers (1Ls) are a class of layered material, which are particularly interesting due to their optoelectronic properties.^9−12^ Optical excitation creates excitons, i.e., bound electron–hole pairs, at the K and K′ direct-bandgap edges,^13,14^ while the strong spin–orbit interaction and broken inversion symmetry leads to coupling of spin and valley degrees of freedom.^15^ Heterostructures comprising two different TMD monolayers can have a type-II band alignment,^16,17^ which localizes electrons in one 1L and holes in the other.^18^ This charge separation results in excitons with a permanent electric dipole moment^19^ and long lifetime (up to 0.2 ms),^20^ due to a reduced overlap of electron and hole wave functions.^21^ While such stacking configurations enable tunability with layer angle and introduce emergent moiré physics,^22^ they are also susceptible to an inhomogeneous potential landscape due to spatial variations in layer separation and twist angle.^23,24^

Janus TMDs (J-TMD) are a class of layered materials^25^ that promise Rashba splitting;^26,27^ piezoelectric response;^28,29^ and long-lived, dipolar excitons^30^ in an intrinsically uniform potential landscape. To form a Janus 1L, a conventional 1L-TMD, such as 1L-WSe_2_, is altered to create 1L-W_Se_^S^ with Se atoms on one face and S atoms on the other, effectively placing a WSe_2_/WS_2_ interface within the 1L. This artificially modified atomic ordering breaks the out-of-plane crystal symmetry and results in an in-built electric field,^31^ which, when experienced by excitons, displaces the electron and hole wave functions.^32^ 1L-Janus were experimentally reported recently in refs (33 and 34). The next steps include the identification and control of exciton charge states in J-TMDs. One challenge is the broad 18-meV photoluminescence (PL) line shape for the narrowest reported emission in J-TMDs, achieved via hexagonal boron nitride (hBN) encapsulation.^35^ The second challenge is feasible integration of J-TMDs into electrically gated devices.

Here, we address the above challenges, and identify neutral and negatively charged exciton transitions in 1L-W_Se_^S^ using reflectance contrast (RC) and PL spectroscopy. To confirm the Janus conversion of a 1L exfoliated from flux-grown WSe_2_ bulk crystal,^68^ we perform Raman and PL spectroscopy over the flake. By encapsulating 1L-W_Se_^S^ in hBN, we are able to measure the narrowest emission (5.9 meV line width) reported to-date from J-TMDs. This spectral narrowing is key to solving the essential challenge of spectrally resolving and assigning the optical transitions to specific exciton charge configurations. Furthermore, we provide physical insights into the excitonic origin of the different optical transitions, by extracting the g factors and trion binding energies. The gate control demonstrated here is a necessary step for future integration into optoelectronic devices and excitonic experiments with J-TMDs.

## Results and Discussion

### Device Characterization

**Figure 1:**
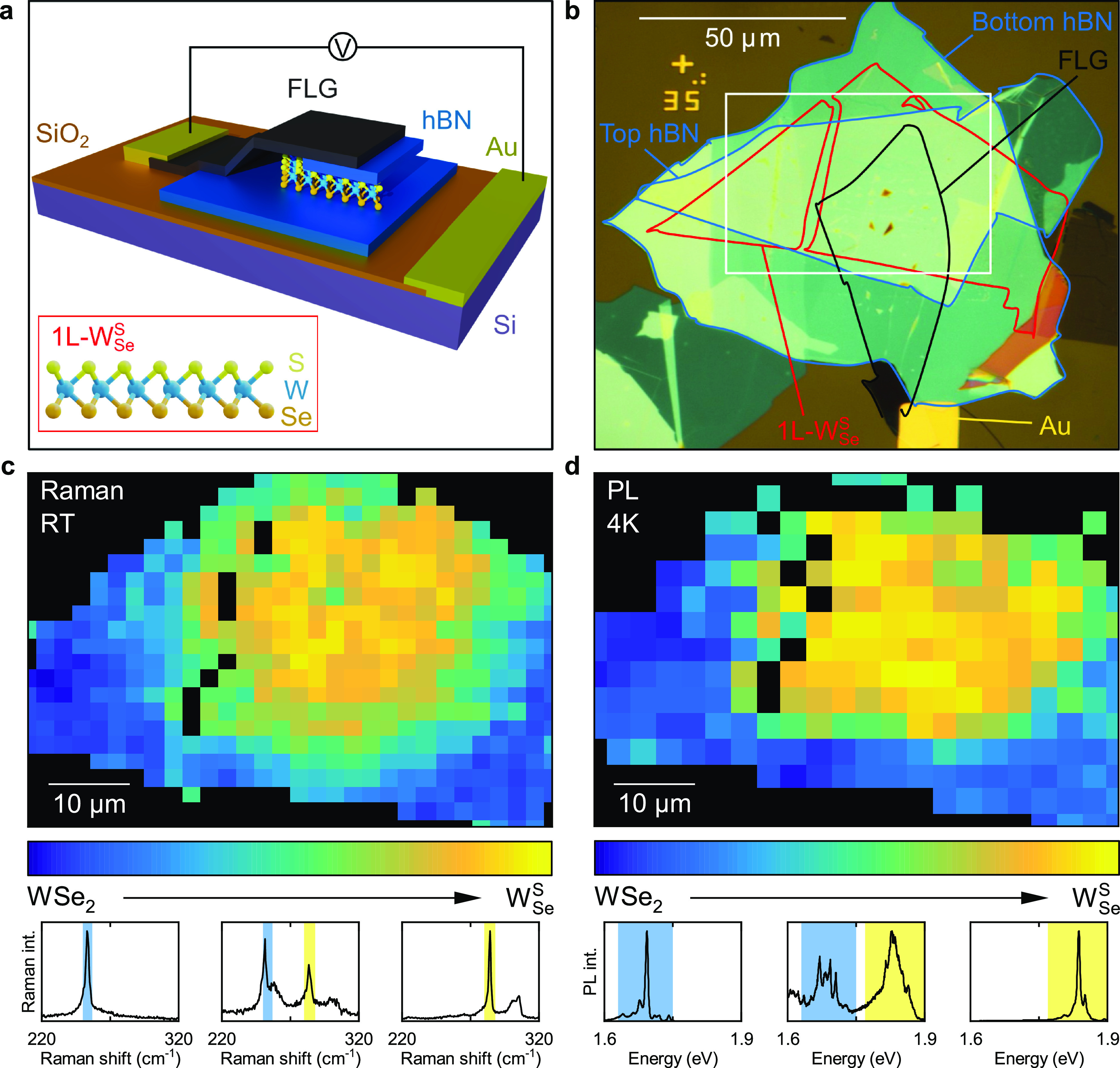
Optical characterization of the Janus 1L-W_Se_^S^ device. (a) Illustration of the device. Janus 1L-W_Se_^S^ (inset) is encapsulated in ML-hBN (blue) and electrically contacted by FLG (black). The device is on a n^+2^ Si (purple)/SiO_2_ (orange) substrate. Au contacts (yellow) allow a voltage to be applied between the FLG and Si. (b) Optical image of the device. 1L-W_Se_^S^ is outlined in red, the top and bottom ML-hBN in blue, and FLG in black. (c) Raman map of the device, in the region highlighted by the white box in (b), acquired at room temperature using 2.33 eV optical excitation. The color coding shows the relative intensity between the 1L-WSe_2_*E*′ + *A*_1_^′^ Raman mode (254 cm^–1^), with 100% in blue, and the Janus 1L-W_Se_^S^*A*_1_^1^ Raman mode (284 cm^–1^), with 100% in yellow. Regions with no 1L-TMD are shown in black. The arrow indicates conversion from 1L-WSe_2_ to 1L-W_Se_^S^. Raman spectra from unconverted, partially converted, and fully converted locations are shown below the Raman map, with the color shading indicating the Raman modes above. (d) PL map of the device in the region highlighted by the white box in (b), acquired at 4 K using 2.33 eV optical excitation. The color coding shows the relative integrated PL emission intensity between the 1L-WSe_2_ (1.63 to 1.75 eV), with 100% in blue, and Janus 1L-W_Se_^S^ (1.77 to 1.91 eV), with 100% in yellow, spectral bands. Regions with no 1L-TMD are shown in black. The arrow indicates conversion from 1L-WSe_2_ to 1L-W_Se_^S^. Representative normalized PL spectra from unconverted, partially converted, and fully converted locations are shown below the PL map, with the color shading indicating the spectral bands above.

Figure 1a is an illustration of one of our Janus devices. The doped Si substrate is used as a back gate, separated from the 1L-Janus by SiO_2_ and multilayer hBN (ML-hBN). A parent 1L-WSe_2_ is exfoliated and subsequently transfered onto the ML-hBN. The 1L-WSe_2_ is then converted into a Janus 1L-W_Se_^S^, with Se atoms on the bottom and S atoms on the top, by following a room-temperature in-situ conversion technique (see Methods and Supplementary Notes S1, S2).^36,37^ An additional ML-hBN transferred on top of the converted 1L-W_Se_^S^ encapsulates the flake, and a top gate of few-layer graphene (FLG) electrically contacts the 1L-W_Se_^S^. Figure 1b shows an optical microscope image of the device, where the 1L-W_Se_^S^ is outlined in red, the bottom and top hBN in blue, and the FLG in black.

Figure 1c shows a Raman spectroscopy map of the device, acquired at room temperature using 2.33 eV optical excitation, in the region highlighted by the white box in Figure 1b. The color code indicates the relative intensity between the characteristic 1L-WSe_2_*E*′ + *A*_1_^′^ Raman mode (blue)^38^ and the Janus 1L-W_Se_^S^*A*_1_^1^ Raman mode (yellow),^39^ with representative Raman spectra from regions with different degrees of Janus conversion shown below the Raman map (see S3). The Raman spectra from the large region (∼400 μm^2^) of fully converted Janus 1L-W_Se_^S^ evidence that the converted region is not a disordered alloy.^36,39,40^

Figure 1d shows a PL map, acquired at a temperature of 4 K using 2.33 eV optical excitation, in the same region of the device as in Figure 1c. Similar to Figure 1c, the color code shows the relative PL emission intensity between the distinct 1L-WSe_2_ (blue) and Janus 1L-W_Se_^S^ (yellow) spectral bands.^36,41^ The PL map correlates with the Raman map in Figure 1c, which validates our assignment of the Janus 1L-W_Se_^S^ spectral band. Therefore, we focus on the exciton emission in the spatial region of full Janus conversion.

### Identification of the Neutral Exciton

**Figure 2:**
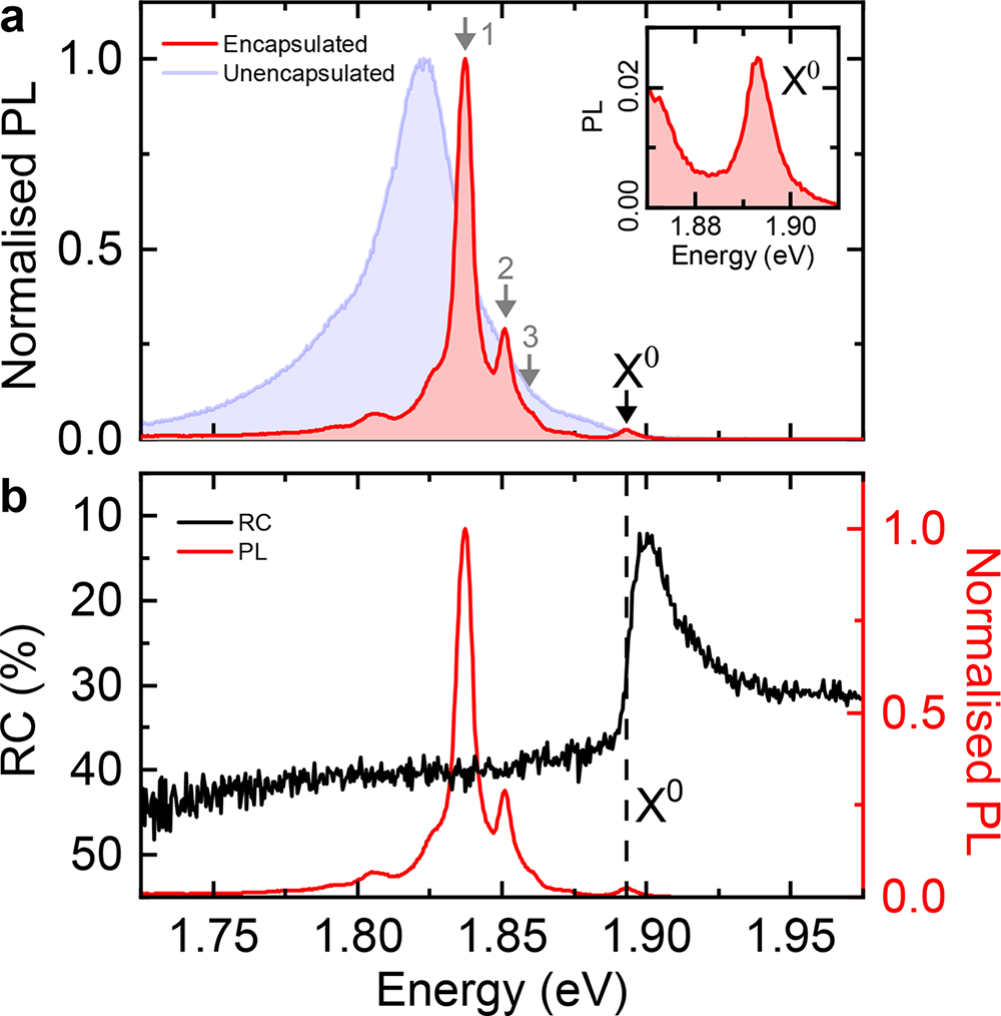
Photoluminescence and reflectance contrast spectra of hBN-encapsulated 1L-W_Se_^S^. (a) PL spectrum from the encapsulated 1L-W_Se_^S^ device (red curve) compared to the PL spectrum from unencapsulated 1L-W_Se_^S^ (blue curve). The spectra are normalized to the same peak height. The peaks labeled 1, 2, and 3 are present across the device. The inset shows the magnified PL spectrum around X^0^. (b) RC spectrum (black curve, left axis) from the encapsulated 1L-W_Se_^S^ device compared to the PL spectrum at the same location (red curve, right axis). The black dashed line denotes the X^0^ transition energy, 1.893 eV. All spectra were acquired at 4 K, in the neutral-doping regime, and the PL spectra under 2.33 eV excitation.

Encapsulation in hBN reduces the line widths of PL peaks in conventional 1L-TMDs,^42−44^ thus allowing for the identification of excitonic species.^45,46^Figure 2a compares a representative PL spectrum at 4 K from our ML-hBN encapsulated 1L-W_Se_^S^ device (red curve) to the spectrum from unencapsulated 1L-W_Se_^S^ on a Si/SiO_2_ substrate (blue curve). The unencapsulated 1L-W_Se_^S^ has a broad spectrum, with a full width at half-maximum (FWHM) on the order 30 meV, on par with the narrowest line width reported to-date for unencapsulated Janus TMDs.^37^ In contrast, encapsulation with hBN allows us to resolve multiple spectral features with significantly reduced line widths (<10 meV).

The peaks labeled 1, 2, 3, and X^0^ are present in the 1L-W_Se_^S^ PL spectra across the whole device (see S3), indicating that these arise from intrinsic excitonic transitions. Since the highest-energy PL peak in both 1L-WSe_2_ and 1L-WS_2_ stems from neutral excitons,^42^ the peak at 1.893 eV is a likely candidate for the neutral exciton, X^0^, in 1L-W_Se_^S^. To verify this, we directly probe excitonic absorption resonances using RC spectroscopy (see Methods).^14^

Figure 2b shows a RC spectrum from our 1L-W_Se_^S^ device (black curve) and the PL spectrum from the same location (red curve). The RC signal shows a strong feature at 1.893 eV, which confirms our assignment of X^0^. The lowest observed PL FWHM of the Janus X^0^ transition is 5.9 meV in our device, the lowest reported to date. The X^0^ transition is present in both PL and RC across the fully converted Janus region (see S3), with an average PL transition energy of 1.890(1) eV and an average FWHM of 8.4(4) meV over 11 measured locations.

Power-dependent PL measurements (see S4) provide further evidence that X^0^ is the neutral exciton transition as its intensity scales linearly with power over the measured range 15 nW to 50 μW (corresponding to 3 Wcm^–2^ to 10^4^ Wcm^–2^). We note that in the spectral range 1.750 to 1.825 eV we also observe PL peaks with linear power dependences at low power and that saturate in the range 50 to 500 nW (10 to 100 Wcm^–2^). This suggests the presence of localized defects displaying quantum light emission.^20,47,48^

Density functional theory (DFT) calculations of the 1L-W_Se_^S^ band structure (see S5) show that, similar to conventional W-based TMDs (1L-WSe_2_ and 1L-WS_2_),^49−51^ 1L-W_Se_^S^ is direct-bandgap at the K points, with a spin ordering such that the upper valence band is opposite in spin to the lower spin-split conduction band. The spin ordering in the conduction band allows for both a negatively charged intervalley trion (X_inter_^–^), with the two electrons in different valleys, and an intravalley trion (X_intra_^–^), with the two electrons in the same valley. By combining DFT and quantum Monte Carlo calculations we predict the binding energies of the Coulomb-exchange split X_inter_^–^ and X_intra_^–^ to be 26 and 32 meV, respectively, relative to the neutral exciton in free-standing 1L-W_Se_^S^.

### Voltage-Controlled Generation of Charged Excitons

**Figure 3:**
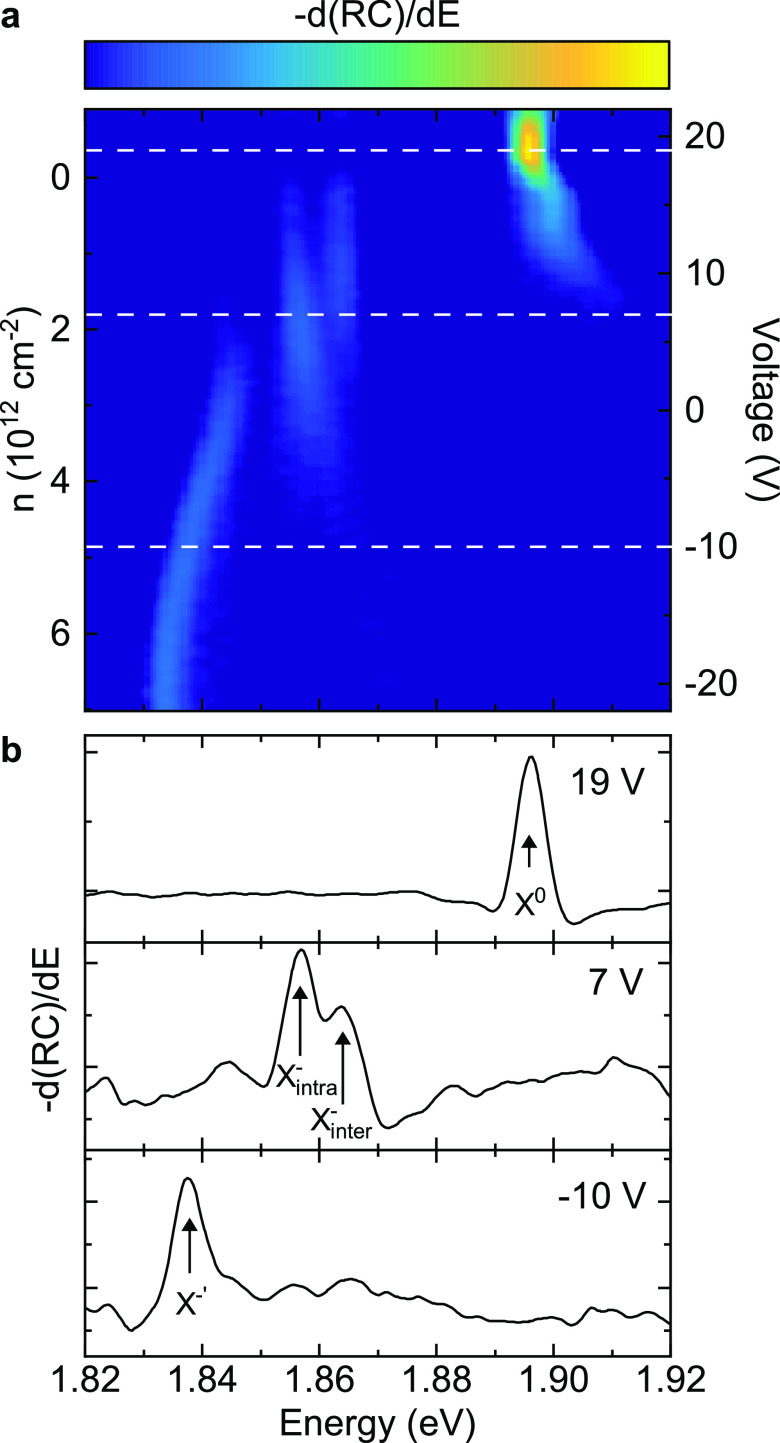
Charge dependence of the reflectance contrast spectrum of 1L-W_Se_^S^. (a) RC derivative with varying electron doping density *n* (left axis) and applied voltage (right axis) at 4 K. (b) RC derivative spectra at the voltages corresponding to the dashed white linecuts in panel (a) at 19, 7, and −10 V. The excitonic transitions X^0^, X_inter_^–^, X_intra_^–^, and X^–′^ are labeled.

To measure the charged excitonic transitions of 1L-W_Se_^S^, we tune its doping by applying a voltage *V* between the 1L-W_Se_^S^ and the Si substrate. Figure 3a shows the RC derivative signal as we vary the doping density, *n* (Methods). Similar doping dependence is observed on a second device (see S2). In the operational range of voltages, only the *n*-doped regime is accessible, due to an intrinsic *n*-doping ∼3 × 10^12^ cm^–2^. The previously identified X^0^ transition, here at 1.896 eV, dominates the RC signal between +21 to +17 V, corresponding to charge neutrality. As we decrease the voltage, and *n*-dope the 1L-W_Se_^S^, lower energy transitions appear, which are analogous to the transitions observed in the *n*-doped regime for 1L-WSe_2_.^52−54^

Figure 3b presents the RC derivative at 19, 7, and −10 V. The neutral exciton, X^0^, is shown in the line cut at 19 V. Between +17 to +5 V, we see a doublet, which we identify as X_inter_^–^ and X_intra_^–^ in the line cut at 7 V with peaks at 1.864 and 1.857 eV. We find an average binding energy relative to X^0^ of 33.4(5) meV and 39.9(3) meV for X_inter_^–^ and X_intra_^–^, respectively, over seven measured locations. We attribute the difference in binding energies of these trions compared to our calculations to a difference in dielectric environment caused by ML-hBN encapsulation.^55^ The exchange splitting between the negative trion transitions of 6.4(6) meV is in good agreement with our calculations.

At increased *n*-doping, below 5 V, the X_inter_^–^ and X_intra_^–^ peaks vanish and a single peak, labeled X^–′^ in the linecut at −10 V in Figure 3b, dominates the derivative of the RC spectrum. The X^–′^ peak initially appears at 1.845 eV and redshifts by 10 meV between +5 and −17 V. An excitonic transition with a similar doping dependence has previously been observed in 1L-WSe_2_^53,54,56^ and attributed to excitons bound to intervalley plasmons.^56,57^ We expect this peak in 1L-W_Se_^S^ to be similar in origin, due to the similarity in its behavior with the transition observed in 1L-WSe_2_.

### Magnetic-Field Dependence of Janus Excitons

**Figure 4:**
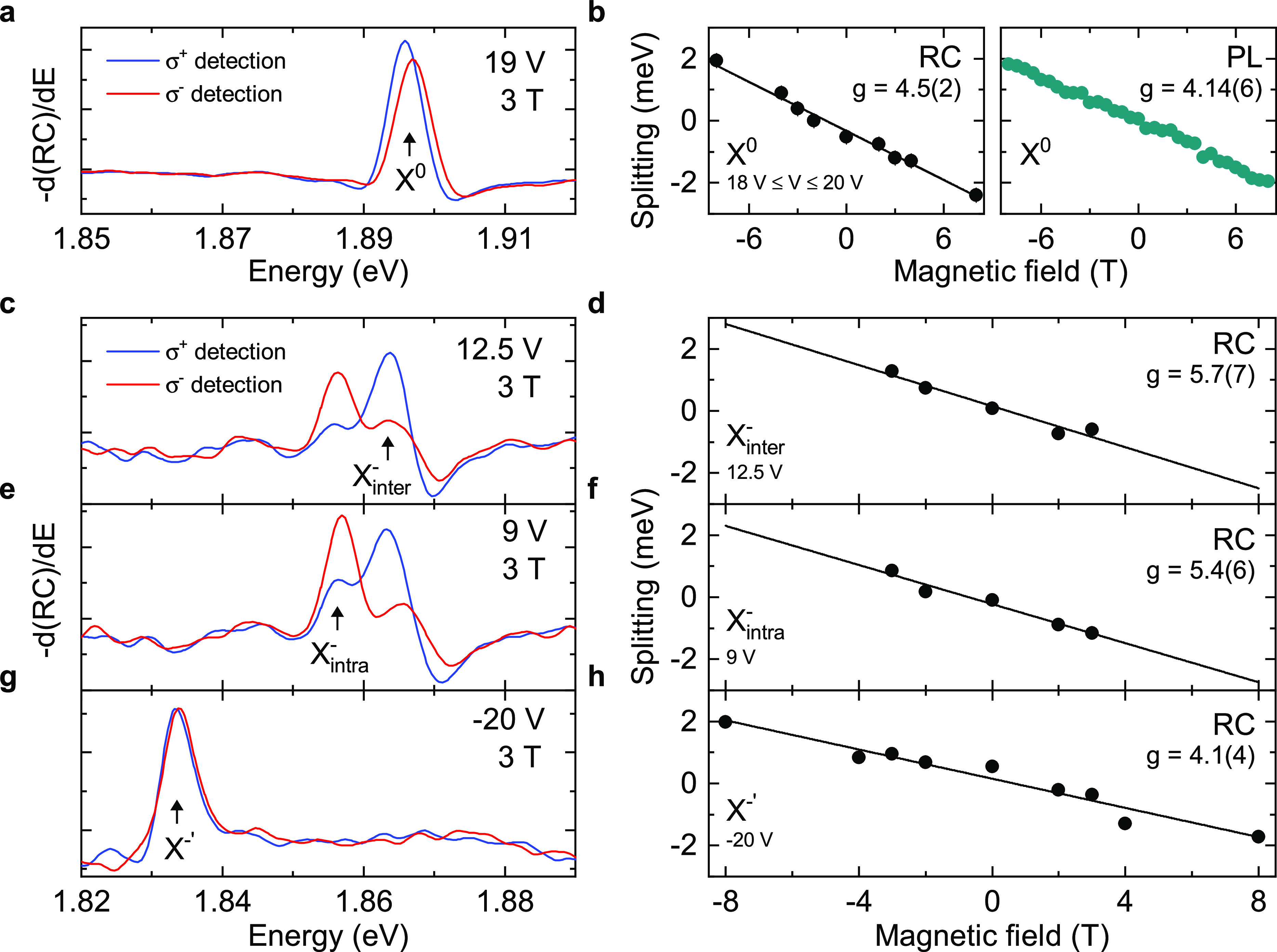
Magnetic field dependence of the excitonic complexes in 1L-W_Se_^S^. (a) RC derivative spectrum with σ^+^ (blue) and σ^–^ (red) polarized collection at *B* = 3 T for X^0^ (at 19 V). (b) The energy splitting Δ*E* between X^0^ peaks in σ^+^ and σ^–^ detected light as a function of magnetic field. The left panel shows the average splitting measured with RC in the neutral regime (splitting averaged between 18 to 20 V at each magnetic field). The right panel shows the splitting of X^0^ measured with PL. The solid curve is a linear fit to Δ*E* = −*g*μ_B_*B*, and the g factors are displayed for both RC and PL. (c) Same measurement as in (a) but for *X*_inter_^–^ at 12.5 V. (d) Δ*E* as a function of magnetic field for *X*_inter_^–^ at 12.5 V. (e,f) Same as in (c) and (d) but for *X*_intra_^–^ at 9 V. (g,h) Same as in (c) and (d) but for the X^–′^ peak at −20 V. All measurements were carried out at 4 K.

We next probe the exciton g factors by applying an out-of-plane magnetic field, *B*, and measuring the Zeeman energy splitting of the exciton transitions. We send unpolarized light to the device and detect the RC spectra with both σ^+^ and σ^–^ circular polarizations. The left-aligned panels (a, c, e, and g) in Figure 4 display the RC derivative spectra for each excitonic transition measured at *B* = 3 T magnetic field, with the right-circular (σ^+^) and left-circular (σ^–^) polarizations shown by the blue and red curves, respectively. The splitting Δ*E* as a function of *B* is shown in the right-hand panels (b, d, f, and h) of Figure 4. Linear fits give the magnitude of the exciton transition g factors, where Δ*E* = −*g*μ_B_*B* (μ_B_ = 58 μeV T^–1^ is the Bohr magneton).

Figure 4a presents the RC derivative spectra for X^0^ at 3 T, showing a well-resolved splitting. Figure 4b shows Δ*E* for X^0^ as a function of magnetic field, for both RC and PL. From the linear fit we extract similar g factors of 4.5(2) and 4.14(6) for RC and PL, respectively. For conventional 1L-TMDs, g factors ∼4 have typically been assigned to bright excitons in the K and K′ valleys, with valley, orbital, and spin contributing to the magnetic moment.^58,59^ The measured g factors are consistent with 1L-W_Se_^S^ having a direct-bandgap at the K points.

The g factors of the negatively charged trions depend strongly on doping, ranging from 3 to 13 for voltages from 8 to 14 V (see S6). For conventional 1L-TMDs, a similar dependence and resulting trion g factors greater than 4 have been attributed to many-body interactions with the Fermi sea of electrons.^60,61^ We expect a similar origin of the observed doping-dependent trion g factor in 1L-W_Se_^S^. Figure 4c–f shows RC derivative spectra and splittings as a function of magnetic field for the negative trions at example voltages. We find example g factors of 5.7(7) for X_inter_^–^ and 5.4(6) for X_intra_^–^ at the voltages presented. The X_inter_^–^ and X_intra_^–^ transitions additionally show evidence of the thermalization of the excess charge, as observed in conventional W-based 1L-TMDs.^45,59,62^ Beyond ∼3 T, this leads to only a single polarization being observable for each negative trion.

Figure 4g shows the polarization-resolved RC derivative spectrum for the X^–′^ transition at 3 T. Figure 4h displays the RC splitting of X^–′^ as a function of magnetic field, which gives a g factor ∼ 4.1(4), consistent with the interpretation of X^–′^ as the exciton bound to intervalley plasmons and dressed by many-body interactions.^53,54,56^

## Conclusions

We identified several excitonic complexes in Janus 1L-W_Se_^S^: X^0^, X_inter_^–^, X_intra_^–^, and X^–′^ and measured their g factors by integrating a hBN encapsulated 1L-W_Se_^S^ into a charge-control device. Integrating J-TMDs into vertical heterostructures is key for the future development of nanoscale optoelectronic devices,^63,64^ while resolving few-meV exciton line widths and identifying the exciton spectrum determines the suitability of J-TMDs for sensing.^65,66^ Future work includes identifying the transitions that give rise to the as-yet unidentified PL peaks as well as measuring the excitonic spectrum in the positively doped regime. An immediate next step is measuring the out-of-plane electric dipole moment of excitons in 1L-W_Se_^S^ by applying an out-of-plane electric field in a capacitor-like device structure. The predicted permanent electric dipole moment of ∼0.2 D for the Janus X^0^^31,67^ means that the resulting Stark shift ∼4 meV at 1 V/nm would be resolved with our ∼6 meV line widths.

## Methods

### Fabrication

We build our device by following a multistep process: first, the bottom ML-hBN is mechanically exfoliated onto a Si/SiO_2_ (90 nm oxide thickness) substrate. Second, a parent 1L-WSe_2_ is mechanically exfoliated from a flux-zone grown^68^ bulk WSe_2_ crystal and deposited on the bottom ML-hBN by polydimethylsiloxane (PDMS) transfer. Third, the 1L-WSe_2_ undergoes AFM flattening^69^ and subsequent conversion to a Janus 1L-W_Se_^S^ by using the selective epitaxial atomic replacement (SEAR) method,^36^ while recording time-resolved Raman spectroscopy measurements in-situ to achieve deterministic conversion.^37^ Fourth, the top ML-hBN and FLG are sequentially deposited on the 1L-W_Se_^S^ by PDMS transfer, with annealing to 150 °C and AFM flattening after each layer is deposited. The FLG is mechanically exfoliated from graphite sourced from HQ Graphene. Fifth, Au contacts are deposited using standard electron-beam lithography procedures.

AFM topography (Bruker Icon) is used to confirm the layer thicknesses, and Raman spectroscopy (Horiba LabRam Evolution) is used to characterize the various constituents of the heterostructure, along with confirming the conversion from 1L-WSe_2_ to 1L-W_Se_^S^ (see S1).

### Optical Measurements

All 4 K measurements are taken in a closed-cycle cryostat (AttoDRY 1000 from Attocube Systems AG), equipped with an 8 T superconducting magnet.

Excitation and collection light pass through a home-built confocal microscope in reflection geometry, with a 0.81 numerical aperture (NA) apochromatic objective (LT-APO/NIR/0.81 from Attocube Systems AG). The PL measurements use continuous-wave excitation from a 2.33 eV laser (Ventus 532 from Laser Quantum Ltd.), with the excitation powers measured on the sample and the optical intensity calculated from the optical spot size given by the 0.81 NA. The PL signal is sent to a 150-line grating spectrometer (Princeton Instruments Inc.).

The RC measurements use broadband light (Thorlabs mounted LED M660L4, nominal wavelength 660 nm, FWHM 20 nm). The reflected light is collected in the confocal microscope discussed above and the spectra are recorded on the same 150-line grating spectrometer as for PL. RC is calculated by comparing the spectrum reflected from the heterostructure in a region with the 1L-W_Se_^S^, *R*, and without 1L-W_Se_^S^, *R*_0_. RC as a function of emission energy *E* is then given by
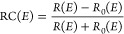


The negative derivative of the RC spectrum, −d(*RC*)/d*E*, highlights the excitonic transitions and suppresses the RC background.^53,70,71^ To obtain the derivative RC spectrum, we first smooth the raw RC spectrum using a spline fit and then take the derivative of the resultant spline.

### Gate-Voltage to Layer-Doping Conversion

The doping density *n* (charge per unit area) is calculated from the applied voltage *V* (Keithley 2400 SMU), by using the gate capacitance, *C*
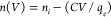
The intrinsic doping, *n*_*i*_, is the doping density at zero applied voltage, and the magnitude of the electron charge is *q*_*e*_ = 1.6 × 10^–19^ C.

The voltage is applied across both the ML-hBN and SiO_2_ and the gate capacitance can be derived by combining the dielectric layers of ML-hBN and SiO_2_ in series
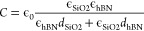
The relative dielectric constants of SiO_2_ and hBN are ϵ_SiO2_ = 3.9^72^ and ϵ_hBN_ = 3.8,^73^ respectively. ϵ_0_ = 8.85 × 10^12^ Fm^–1^ is the vacuum permittivity.

The thickness of SiO_2_ is *d*_SiO2_ = 90 nm and that of hBN is *d*_hBN_ = 27 nm (see S1). The intrinsic doping density is *n*_*i*_ = 3 × 10^12^ cm^–2^, determined by setting the doping density to *n* = 0 when the reflectance contrast signal from the neutral exciton vanishes (17 V),^53^ where positive *n* indicates electron doping.

## Supporting Information Available

Device characterization; Gate-dependent measurements in device 2; Spectral mapping and homogeneity; Power-dependent measurements; Band structure calculations; Magnetic-field dependent measurements (PDF)

## Notes

This work has previously been submitted to a preprint server:^74^ Feuer, M. S. G.; Montblanch, A. R. P.; Sayyad, M.; Purser, C. M.; Qin, Y.; Alexeev, E. M.; Cadore, A. R.; Rosa, B. L. T.; Kerfoot, J.; Mostaani, E.; Kalba, R.; Kolari, P.; Kopaczek, J.; Watanabe, K.; Taniguchi, T.; Ferrari, A. C.; Kara, D. M.; Tongay, S.; Atature, M. Identification of exciton complexes in a charge-tunable Janus WSeS monolayer. *arXiv*, October 13, 2022, arXiv:2210.06930. DOI: 10.48550/arXiv.2210.06930 (accessed March 22, 2023).
