# Supplementary material for: Identification of Exciton Complexes in Charge-Tunable Janus WSeS Monolayers
Source: ACS Nano. 2023 Apr 14;17(8):7326–34. doi: 10.1021/acsnano.2c10697 (PMC10134503; doi:10.1021/acsnano.2c10697)
Supplement: Supplementary file 1 — nn2c10697_si_001.pdf [file nn2c10697_si_001.pdf]

Supporting Information for:
"Identification of exciton complexes in a charge-tuneable Janus W^{S}_{Se} monolayer"

Matthew S. G. Feuer,[1,*] Alejandro R.-P. Montblanch,[1,*] Mohammed Y. Sayyad,[2,*] Carola M. Purser,[1,3] Ying Qin,[2] Evgeny M. Alexeev,[3,1] Alisson R. Cadore,[3] Barbara L. T. Rosa,[3] James Kerfoot,[3] Elaheh Mostaani,[3] Radosław Kalęba,[1] Pranvera Kolari,[2] Jan Kopaczek,[2] Kenji Watanabe,[4] Takashi Taniguchi,[5] Andrea C. Ferrari,[3] Dhiren M. Kara,[1] Sefaattin Tongay,[2,†] and Mete Atatüre[1,‡]

[1] *Cavendish Laboratory, University of Cambridge, 19 J. J. Thomson Ave., Cambridge, CB3 0HE, UK*
[2] *Materials Science and Engineering, School for Engineering of Matter, Transport and Energy, Arizona State University, Tempe, Arizona, 85287, USA*
[3] *Cambridge Graphene Centre, University of Cambridge, 9 J. J. Thomson Ave., Cambridge, CB3 0FA, UK*
[4] *Research Center for Functional Materials, National Institute for Materials Science, 1-1 Namiki, Tsukuba 305-0044, Japan*
[5] *International Center for Materials Nanoarchitectonics, National Institute for Materials Science, 1-1 Namiki, Tsukuba 305-0044, Japan*

CONTENTS

Supplementary Note S1. Device characterisation 1

Supplementary Note S2. Gate-dependent measurements in device 2 4

Supplementary Note S3. Spectral mapping and homogeneity 5

Supplementary Note S4. Power-dependent measurements 7

Supplementary Note S5. Band structure calculations 9

Supplementary Note S6. Magnetic-field dependent measurements 10

References 11

Supplementary Note S1. DEVICE CHARACTERISATION

Supplementary Figure 1 shows the characterisation of the layer thicknesses by atomic force microscope (AFM) topography (Bruker Icon). Supplementary Fig. 1a and b show the height profiles for the bottom and top multilayer hexagonal boron nitride (ML-hBN), respectively. Step height analysis was performed in Gwyddion, by obtaining the average height across an appropriate line cut (black circles) and fitting the 'smooth bent step' function to extract the layer thickness from the step height [1]. This gives the bottom and top ML-hBN thickness as 27.2(2) nm and 12.1(3) nm respectively, as shown by the red lines in Supplementary Figs. 1b and 1c. Supplementary Fig. 1c shows the height profile for the few layer graphene (FLG), which gives the FLG thickness as 6.3(2) nm.

The Janus monolayers are converted from their parent TMD using selective epitaxial atomic replacement (SEAR), with optimised protocols to yield highly ordered crystalline structures [2, 3]. We verify that the SEAR conversion process produces ordered Janus structures by using high resolution scanning transmission electron microscopy. Supplementary Fig. 2 plots the High Angle Annular Dark Field (HAADF) of the monolayer before and after the SEAR conversion. Prior to conversion, the Rutherford scattering intensity from the partially screened atomic nucleus of the Se atom scales as $Z^{1.7}$ [4]. Therefore, 1L-WSe_2 shows a bright contribution from the two Se atoms ($I_{\text{Chalcogen}} = 2Z_{\text{Se}}^{1.7}$). After conversion to the Janus structure, the scattering intensity from the chalcogen atoms decreases due to the presence of lighter S atoms ($I_{\text{Chalcogen}} = Z_{\text{Se}}^{1.7} + Z_{\text{S}}^{1.7}$). Furthermore, the intensity distribution is highly ordered, thus ruling out the possibility of formation of non-Janus ordering or alloying.

* These authors contributed equally to this work.
† sefaattin.tongay@asu.edu
‡ ma424@cam.ac.uk

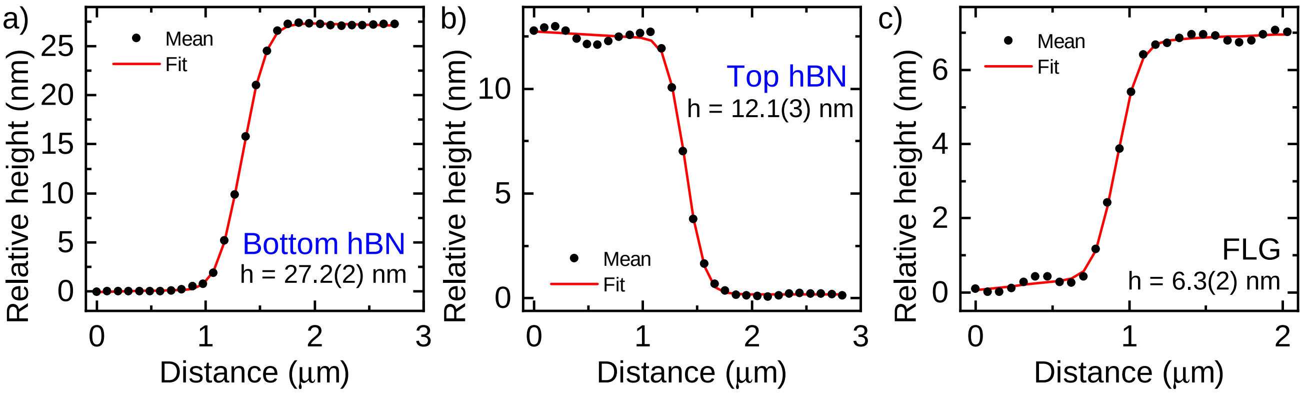

Supplementary Figure 1. AFM height profiles of the device. **(a)** Height profile for the bottom ML-hBN, obtained as the average height (black circles) across a line cut. The red line is a fit to a 'smooth bent step', which gives the step height h. **(b)** Height profile for the top ML-hBN, obtained as the average height (black circles) across a line cut. The red line is a fit to a 'smooth bent step', which gives the step height h. **(c)** Height profile for the FLG, obtained as the average height (black circles) across a line cut. The red line is a fit to a 'smooth bent step', which gives the step height h.

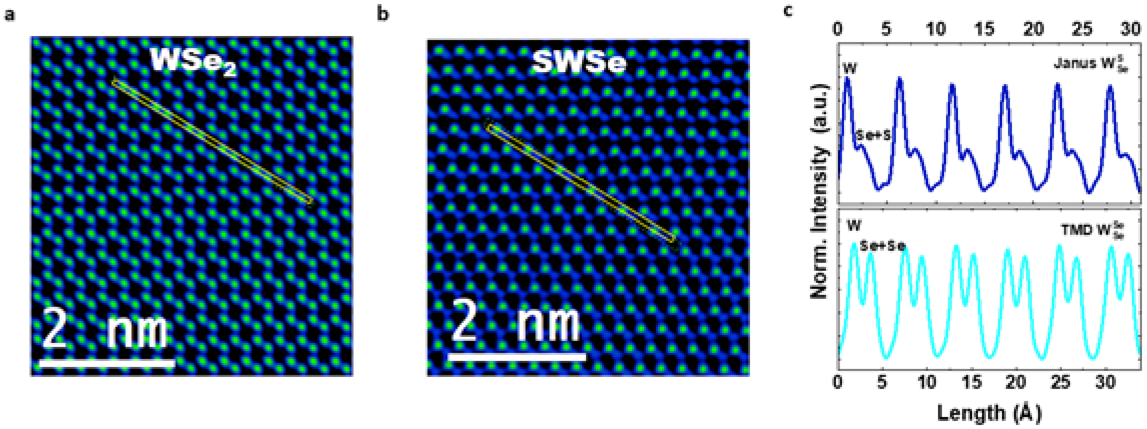

Supplementary Figure 2. High Angle Annular Dark Field microscopy. **(a)** From monolayer WSe_2 and **(b)** JanusW^S_{Se}. **(c)** Line intensity profiles from a and b, showing a highly ordered reduction in the Rutherford scattering intensity due to the lighter chalcogen atom of sulphur.

We perform room-temperature Raman spectroscopy in a commercial Horiba LabRam Evolution system, using 2.33 eV optical excitation. Supplementary Figure 3a shows the Raman spectra from an un-converted WSe_2 monolayer (1L-WSe_2) region of the device as the black curve and fully converted Janus W^S_{Se} monolayer (1L-W^S_{Se}) region as the red curve. The peak at ~ 253 cm^{-1} from 1L-WSe_2 is assigned to the convoluted $E' + A_1'$ modes, which are degenerate in 1L-WSe_2 [5, 6]. The first-order Raman peaks from 1L-W^S_{Se} are assigned as the E^1 mode at ~ 205 cm^{-1}, the A_1^1 mode at ~ 283 cm^{-1}, the E^2 mode at ~ 331 cm^{-1} and the A_1^2 mode at ~ 421 cm^{-1} [7]. The other Raman peaks present arise from higher-order Raman modes, as discussed in ref. [7]. The distinct Raman modes between 1L-WSe_2 and Janus 1L-W^S_{Se} distinguishes the un-converted 1L-WSe_2 and fully converted 1L-W^S_{Se} regions. Furthermore, the 1L-W^S_{Se} Raman spectra also confirms we have ordered Janus 1L-W^S_{Se} rather than a disordered ternary alloy, which would show the representative Raman peaks of 1L-WSe_2 and 1L-WS_2 [2, 7, 8].

Supplementary Figure 3b shows the Raman spectra from the FLG (black curve) and ML-hBN (red curve). The peak at ~ 1366 cm^{-1} from ML-hBN is assigned to the E_{2g} mode [9, 10]. The G peak from the FLG is seen at ~ 1582 cm^{-1} and the 2D band at ~ 2720 cm^{-1} [11].

The photoluminescence (PL) spectra from the 1L-WSe_2 and 1L-W^S_{Se} regions of the device are compared in Supplementary Figure 4. The left-hand panels (a and b) compare the room-temperature PL spectra, with the spectrum from a ML-hBN-encapsulated un-converted 1L-WSe_2 region of the device in Supplementary Fig. 4a. Supplementary Fig. 4b compares the spectrum from an unencapsulated 1L-W^S_{Se} sample (blue curve), which is directly on a Si/SiO_2 substrate and converted from exfoliated 1L-WSe_2, to the ML-hBN-encapsulated 1L-W^S_{Se} (red curve) region of the

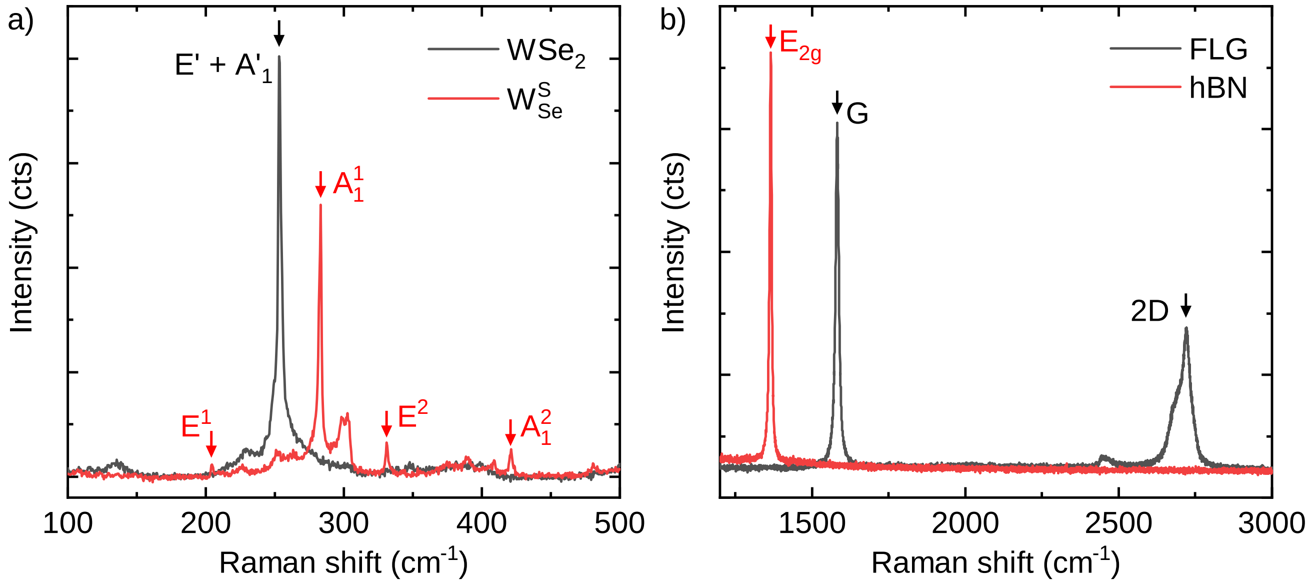

Supplementary Figure 3. Raman spectra from the device, acquired at room temperature using 2.33 eV optical excitation. **(a)** Raman spectra from an un-converted 1L-WSe_2 (black curve) and fully converted Janus 1L-W^{S}_{Se} (red curve) region of the device. The labelled peaks are the 1L-WSe_2 $E' + A'_1$ and the 1L-W^{S}_{Se} first-order Raman modes: E^1, A^1_1, E^2 and A^2_1. **(b)** Raman spectra from the FLG (black curve) and ML-hBN (red curve). The labelled peaks are the G and 2D Raman modes from the FLG and the E_{2g} Raman mode from the ML-hBN.

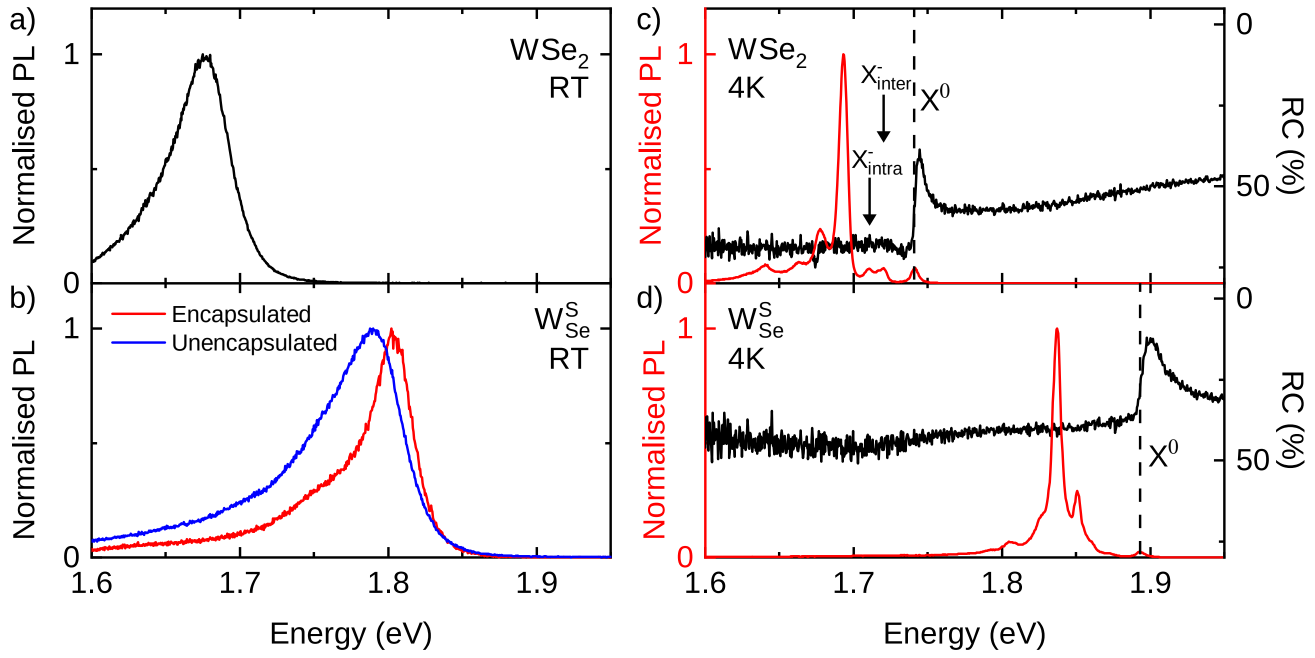

Supplementary Figure 4. Comparison of spectra from 1L-WSe_2 and 1L-W^{S}_{Se}, at room temperature and 4 K. **(a)** Room temperature PL spectrum from an un-converted ML-hBN-encapsulated 1L-WSe_2 region of the device. **(b)** Room temperature PL spectra from Janus 1L-W^{S}_{Se}, comparing the ML-hBN-encapsulated 1L-W^{S}_{Se} device (red curve) with unencapsulated 1L-W^{S}_{Se} (blue curve). Spectra are normalised to the same peak height. **(c)** 4 K PL (red curve, left axis) and RC (black curve, right axis) spectra from a location in the 1L-WSe_2 region of the device. The arrows mark the energy of the intra- and inter-valley trion and the dashed line shows the energy of the neutral exciton, X^0. **(d)** 4 K PL (red curve, left axis) and RC (black curve, right axis) from a location in the encapsulated1L-W^{S}_{Se} region of the device. The dashed line marks the energy of the X^0. All PL spectra were acquired with 2.33 eV excitation.

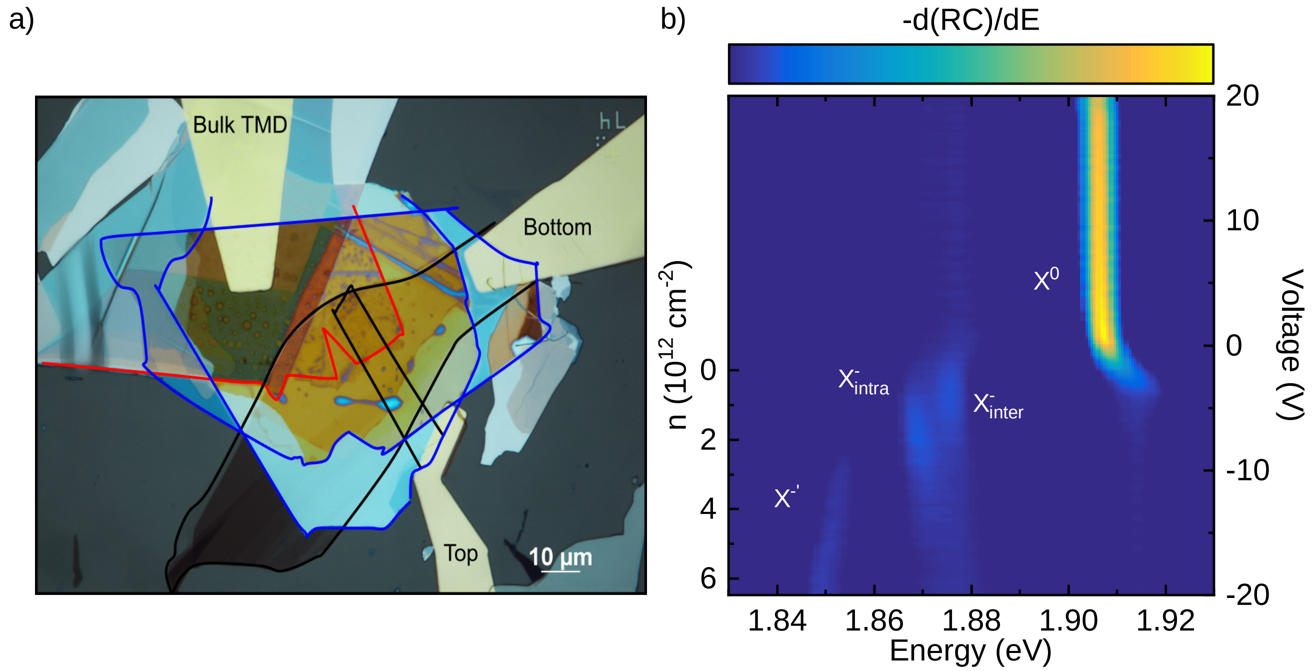

Supplementary Figure 5. Charge dependence of the reflectance contrast for a second 1L-W^{S}_{Se} device. **(a)** Optical image of the second device. The 1L-W^{S}_{Se} is outlined in red, the top and bottom ML-hBN in blue and the top and bottom FLG gates in black. The bulk TMD, bottom FLG and top FLG gates are electrically contacted by gold. **(b)** The derivative of RC with varying electron doping density n (left axis) and applied bottom gate voltage (right axis) at 4 K.

device. The emission peak intensity is spectrally shifted from ∼1.675 eV in 1L-WSe_2 to ∼1.8 eV in 1L-W^{S}_{Se}. Peak widths are estimated from a Gaussian fit to the high energy side of the spectra, which gives ∼50 meV for the unencapsulated 1L-W^{S}_{Se}, ∼42 meV for the encapsulated 1L-WSe_2 and ∼35 meV for the encapsulated 1L-W^{S}_{Se}. The width of the unencapsulated 1L-W^{S}_{Se} emission is similar to the best reported for unencapsulated 1L-W^{S}_{Se} at room temperature [3]. Spectral narrowing is seen in the room temperature 1L-W^{S}_{Se} emission upon encapsulation, to below the width of the emission from the encapsulated 1L-WSe_2 region of the device. The highest peak at ∼ 1.8 eV in the encapsulated 1L-W^{S}_{Se} spectrum likely arises from the recombination of the neutral exciton, as identified in the temperature dependence of ref. [3].

The PL (red curve) and reflection contrast (RC) spectra (black curve) at 4 K are shown in the right-hand panels (c and d) of Supplementary Figure 4, for both the 1L-WSe_2 region of the device in Supplementary Fig. 4c and the 1L-W^{S}_{Se} region in Supplementary Fig. 4d. The PL spectra narrow considerably from room temperature and multiple resolvable peaks can be seen in both regions. In the 1L-WSe_2 region, the PL spectrum is consistent with previous reports [12] and we can identify the the neutral exciton, X^0, at 1.741 eV (width 5 meV); the inter-valley negatively charged trion, X^{-}_{inter}, at 1.720 eV (width 6 meV); and the intra-valley negatively charged trion, X^{-}_{intra}, at 1.710 eV (width 9 meV). The 1L-WSe_2 RC spectrum shows the neutral exciton feature at 1.741 eV, coincidental in energy with the X^0 PL peak. On moving to the 1L-W^{S}_{Se} region, the spectral range of the emission shifts to above ∼ 1.77 eV but the spectral widths remain similar. As discussed in the main text, the highest energy peak is identified as arising from 1L-W^{S}_{Se} X^0 and the RC signal is coincidental with this.

The RC signal from 1L-WSe_2 uses a different light source (Thorlabs M730L5) compared to the RC from 1L-W^{S}_{Se} (Thorlabs M660L4), due to the different spectral range of the transitions.

Supplementary Note S2. GATE-DEPENDENT MEASUREMENTS IN DEVICE 2

We have measured similar results to those discussed in the main text on a second sample (device 2). Supplementary Figure 5a shows the optical image of device 2. This second device consists of a converted region of 1L-W^{S}_{Se} (outlined in red), attached to bulk unconverted WSe_2. The 1L-W^{S}_{Se} is encapsulated by 61 nm of bottom ML-hBN and 43 nm of top ML-hBN (both outlined in blue). A bottom FLG gate is under the bottom ML-hBN and top FLG gate above the top ML-hBN (both outlined in black). Electrical contacts to the bulk TMD, top and bottom FLG gates are provided by gold leads. The device is fabricated using the same polydimethylsiloxane (PDMS) transfer technique as for device

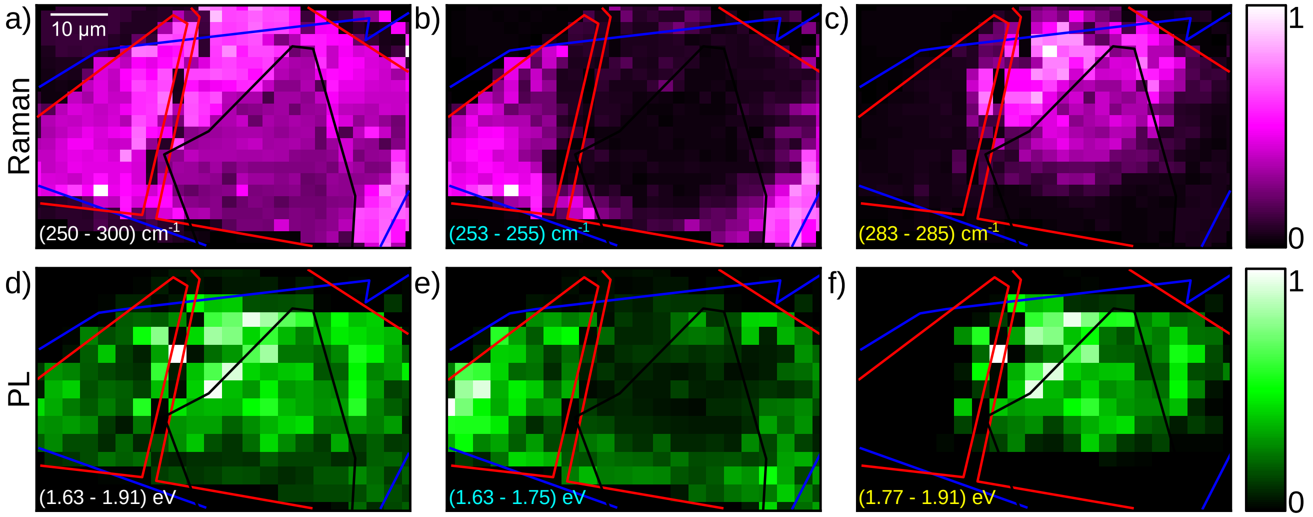

Supplementary Figure 6. Integrated Raman and PL maps from the device. **(a) - (c)** Integrated Raman maps, acquired at room temperature and using 2.33 eV optical excitation. The integration range is over Raman shifts 250 to 300 cm^{-1} in **a**, 253 to 255 cm^{-1} (1L-WSe$_2$ $E' + A_1'$ Raman mode) in **b** and 283 to 285 cm^{-1} (1L-W$^{\mathrm{S}}_{\mathrm{Se}}$ A_1^1 Raman mode) in **c**. **(d) - (f)** Integrated PL maps, acquired at 4 K and using 2.33 eV excitation. The integration range is over the spectral band 1.63 to 1.91 eV in **d**, 1.63 to 1.75 eV (1L-WSe$_2$ spectral band) in **e** and 1.77 to 1.91 eV (1L-W$^{\mathrm{S}}_{\mathrm{Se}}$ spectral band) in **f**. All maps are over the same region of the device as highlighted by the white box in Fig. 1b of the main text. Each panel is normalised to the maximum intensity within the panel. The red outline corresponds to the 1L-W$^{\mathrm{S}}_{\mathrm{Se}}$, the blue outline to the top ML-hBN and the black outline to the FLG.

1 (see Methods) and the Janus conversion uses the same SEAR in-*situ* process [2, 3]. Layer thicknesses are confirmed by AFM topography.

Supplementary Figure 5b shows the RC derivative as we tune the doping density, n, for device 2. The doping density is tuned here by applying a voltage between the TMD and bottom FLG gate and n is calculated as described in Methods, using the 61 nm bottom ML-hBN thickness. We set the band edge $n = 0$ to be where the neutral exciton RC signal vanishes and find the intrinsic doping to be $n_i = 0.7 \times 10^{12}$ cm^{-2}. In the n-doped regime we see the same transitions and behaviour as for device 1 (see Fig. 3 of the main text), namely the neutral exciton X^0, the inter- and intra-valley negative trions (X$^-_{\mathrm{inter}}$ and X$^-_{\mathrm{intra}}$, respectively) and the X$^{-\prime}$ transition. Device 2 has a lower level of intrinsic doping than device 1; however, as we change to positive voltage and attempt to enter the p-doped regime we observe that the neutral exciton persists and thus we are unable to reach the p-doped regime.

We find that the neutral exciton transition X^0 is at 1.907 eV at this location on device 2, which is a 17 meV shift from the average across device 1 and possibly arises from a difference in strain on the device. We see the X$^-_{\mathrm{inter}}$ transition at 1.875 eV and X$^-_{\mathrm{intra}}$ at 1.869 eV. Across four measured locations on this device, we find average trion binding energies of 31.3(5) meV and 37.5(3) meV for X$^-_{\mathrm{inter}}$ and X$^-_{\mathrm{intra}}$, respectively, agreeing with those measured on device 1. This gives an exchange splitting of 6.3(6) meV, agreeing with both our calculation (see Supplementary Note S5) and measurement on device 1. As in device 1, we see the X$^{-\prime}$ transition, which emerges at 1.854 eV and red shifts with increased doping. The doping densities where the various transitions appear in device 2 are also similar to those in device 1. Specifically, the cross-over between negative trions and X$^{-\prime}$ occurs around $(2-3) \times 10^{12}$cm^{-2} in both devices.

Supplementary Note S3. SPECTRAL MAPPING AND HOMOGENEITY

Supplementary Figure 6 presents integrated spectral maps over the device, for both Raman and PL spectroscopy, which are used to calculate the relative intensity maps in Figs. 1c and d of the main text. Supplementary Figs. 6a - 6c show the room-temperature integrated Raman maps of the characteristic Raman modes from 1L-WSe$_2$ and 1L-W$^{\mathrm{S}}_{\mathrm{Se}}$. Supplementary Fig. 6a shows the integrated Raman intensity over the spectral range (250 to 300 cm^{-1}) covering both the 1L-WSe$_2$ and 1L-W$^{\mathrm{S}}_{\mathrm{Se}}$ characteristic modes. Supplementary Fig. 6b shows the integrated intensity from the 1L-WSe$_2$ $E' + A_1'$ Raman mode (253 to 255 cm^{-1}) and Supplementary Fig. 6c shows the integrated intensity from the 1L-W$^{\mathrm{S}}_{\mathrm{Se}}$ A_1^1 Raman mode (283 to 285 cm^{-1}). This shows that the flake has a central region of fully converted 1L-W$^{\mathrm{S}}_{\mathrm{Se}}$, as shown by the yellow region in the relative intensity map of Fig. 1c from the main text.

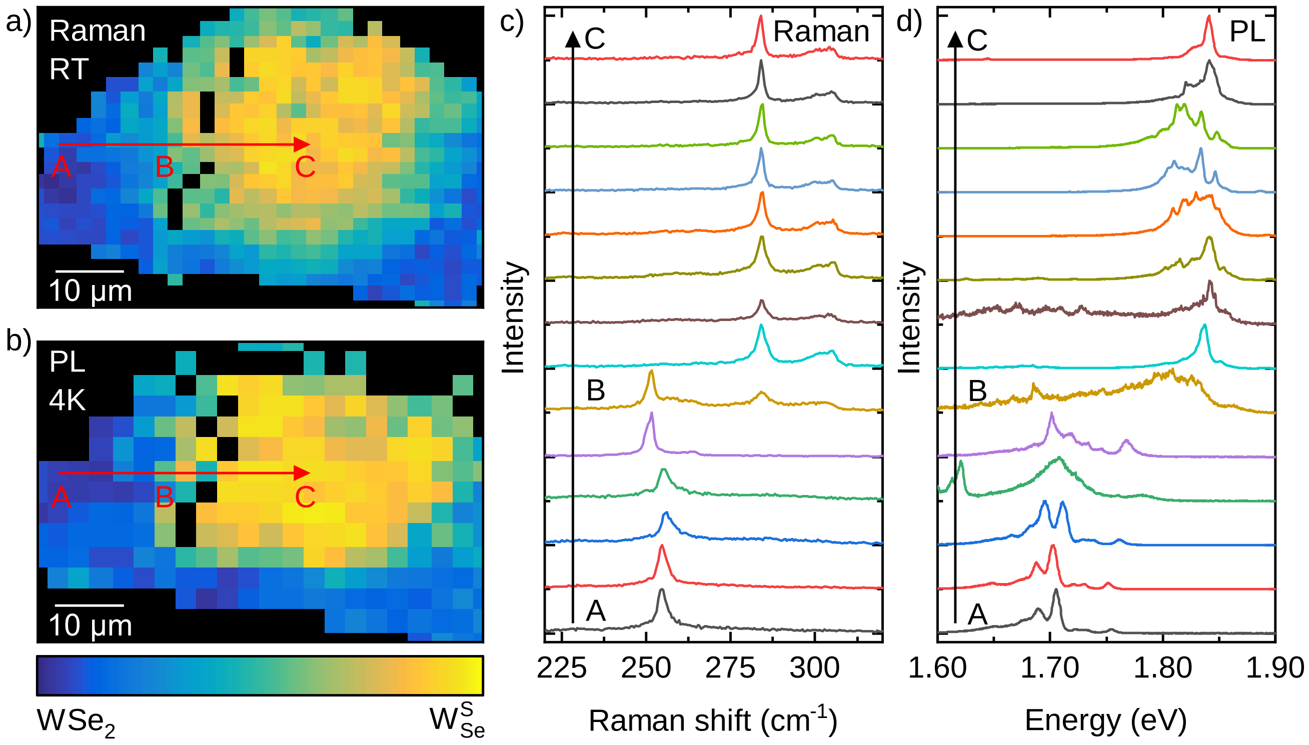

Supplementary Figure 7. Raman and PL spectra across paths in the Raman and PL maps. **(a)** Raman map of the device from Fig. 1c of the main text, acquired at room temperature and using 2.33 eV optical excitation. **(b)** PL map of the device from Fig. 1d of the main text, acquired at 4 K and using 2.33 eV excitation. **(c)** Raman spectra along the red path in **a**, from the un-converted 1L-WSe_2 region (point A), through the partially converted region (point B) to the fully converted 1L-W^{S}_{Se} region (point C). The spectra are vertically offset for clarity. **(d)** PL spectra along the red path in **b**, from the un-converted 1L-WSe_2 region (point A), through the partially converted region (point B) to the fully converted 1L-W^{S}_{Se} region (point C). The spectra are vertically offset for clarity.

Supplementary Figs. 6d - 6f show the PL maps, acquired at 4 K, of the integrated spectral bands associated with 1L-WSe_2 and 1L-W^{S}_{Se}. Supplementary Fig. 6d shows the integrated PL intensity over the spectral band (1.63 to 1.91 eV) covering both the 1L-WSe_2 and 1L-W^{S}_{Se} emission. Supplementary Figs. 6e and 6f show the integrated intensity from the 1L-WSe_2 (1.63 to 1.75 eV) and 1L-W^{S}_{Se} (1.77 to 1.91 eV) spectral bands, respectively. The integrated PL maps show a central region of converted 1L-W^{S}_{Se}, consistent with the integrated Raman maps and shown by the yellow region in the relative intensity map of Fig. 1d from the main text.

Within the relative intensity maps in Figs. 1c and d of the main text, the black pixels correspond to the absence of the monolayer TMD. These pixels have been identified from the experimental data by setting a threshold (5% of the maximum intensity) on the total spectrally integrated intensity of the signal, where values less than this threshold correspond to the absence of the TMD monolayer and hence are coloured black. The outline of the monolayer identified in this way from the experimental data agrees well with the red outline of the monolayer in the optical image (Fig. 1b). The black pixels within the monolayer correspond to the crack in the monolayer flake evident in the optical image (Fig. 1b).

Supplementary Figure 7 shows the variation in Raman and PL spectra across paths of varying Janus conversion. Supplementary Figure 7c and 7d plot the Raman and PL spectra along the paths indicated in Supplementary Figs. 7a and 7b, respectively. Note that these paths were taken on the same region as the one shown in Figs. 1c and 1d from the main text. The paths are along the red lines from the un-converted 1L-WSe_2 region (point A), through the partially converted region (point B), to the fully converted 1L-W^{S}_{Se} region (point C). The partially converted regions show the distinctive Raman modes from both 1L-WSe_2 and 1L-W^{S}_{Se}, along with PL emission in both spectral bands. The un-converted and fully converted regions show the Raman modes corresponding to either 1L-WSe_2 or 1L-W^{S}_{Se}, and PL emission from a single spectral band.

Supplementary Fig. 8 presents the variation in PL and RC spectra at nine locations across the sample in the 1L-W^{S}_{Se} region of the device. Across the sample, we see changes in the relative intensity of the different PL peaks and width of the redder emission tail. However, the general structure of the higher energy peaks persists across different locations, and specifically the peaks labelled in Fig. 2a of the main text (1, 2, 3 and X^0) are identifiable. Across the device,

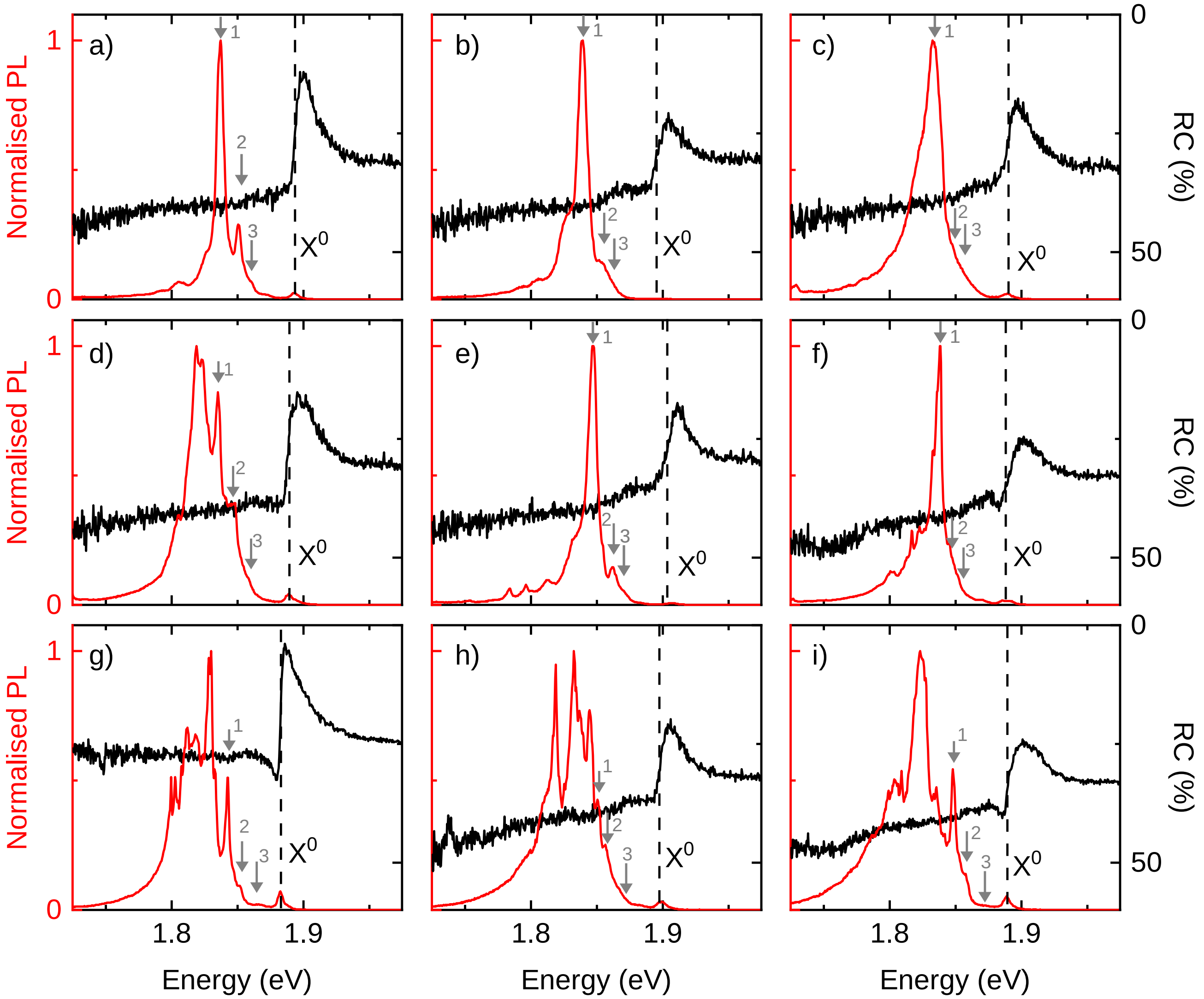

Supplementary Figure 8. PL and RC spectra from 1L-W^{S}_{Se} across different locations. **(a) - (i)** PL (red curve, left axis) and RC (black curve, right axis) spectra at different locations in the 1L-W^{S}_{Se} region of the device. Each panel shows the PL and corresponding RC spectra at a single location. All spectra were acquired at 4 K, and the PL spectra with 2.33 eV excitation. Panel **a** appears as Fig. 2b of the main text.

the RC feature is coincidental with the X^0 peak in PL but with contrast dependent on location. As discussed in the main text, calculating statistics across the device gives the average X^0 PL peak energy as 1.890(1) eV and FWHM as 8.4(4) eV. A similar variation in PL spectra is also seen in the best WSe_2 samples [12, 13] and likely arises from a variation in strain and fabrication inhomogeneity.

Supplementary Note S4. POWER-DEPENDENT MEASUREMENTS

Supplementary Figure 9 shows the excitation power dependence of the PL emission from 1L-W^{S}_{Se}, over the full spectral range in Supplementary Fig. 9a and over the spectral range of the neutral exciton in Supplementary Fig. 9b. The spectra remain of a similar shape as the excitation power is changed from 15 nW to 50 μW (corresponding to 3 Wcm^{-2} to 10^4 Wcm^{-2}), except for the appearance of low energy peaks at low powers. The excitation power dependence of the integrated intensity of the resolvable peaks (labelled 1, 2 and X^0) in the high power spectrum are shown in Supplementary Fig. 9c. A power law fit shows that these three peaks follow a linear power law scaling, consistent with assignment of these peaks as arising from free excitonic species [12]. Specifically, the linear power dependence of the X^0 peak provides evidence of it arising from the recombination of the neutral exciton, as discussed

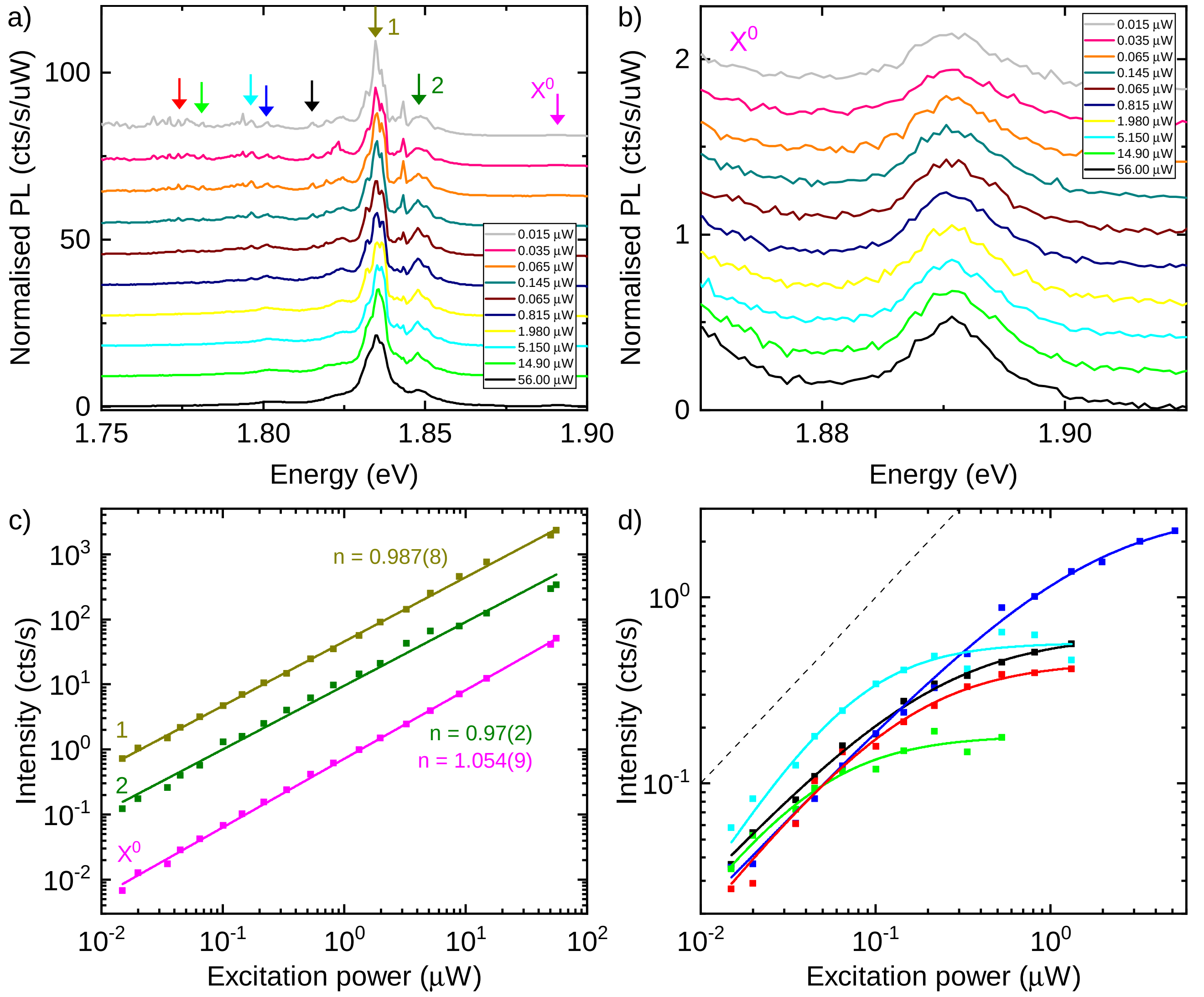

Supplementary Figure 9. Excitation-power PL dependence of the 1L-W$^{S}_{Se}$ emission, acquired at 4 K and using 2.33 eV excitation. **(a)** Power dependence of the PL spectra, normalised by integration time and excitation power. The excitation power is varied between 15 nW (grey curve) to 56 μW (black curve). Spectra are vertically offset for clarity. **(b)** Same as in **a**, but showing only the neutral exciton X^0. **(c)** Excitation power dependence of the PL intensity of the labelled peaks 1, 2 and X^0 from **a** and **b**. These are the resolvable peaks at high power. The data is plotted on a double logarithmic scale and the solid line is a fit to $I \propto P^n$, with peak intensity I, power P and power law scaling n. All three peaks give linear power law scaling. **(d)** Same as in **c** but for the unlabelled peaks in **a** as indicated by coloured arrows, which appear at low excitation power. The solid line is a fit to $I \propto P^n/(P^n + P^n_{\text{sat}})$, with saturation power P_{sat}. The dashed line shows a linear power scaling $n = 1$. All these peaks show an initially linear power dependence, before their intensity saturates.

in the main text. We see no evidence of a blueshift to these spectral peaks with increasing excitation power, up to the maximum accessible power of ~ 50 μW (10^4 Wcm^{-2}). The power dependence of the intensity of the marked low energy peaks (labelled by coloured arrows) are shown in Supplementary Fig. 9d. In contrast to the higher energy peaks, we see that their intensity follows a linear power scaling at low powers and saturates at powers in the range 50 to 500 nW (10 to 100 Wcm^{-2}). As noted in the main text, this points to the presence of localised defects displaying quantum light emission [14–16].

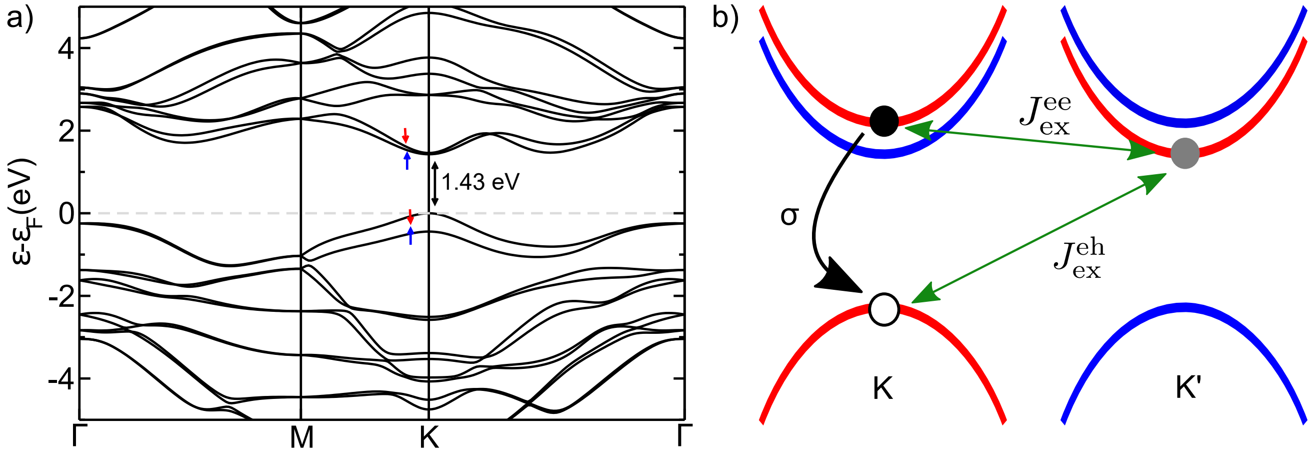

Supplementary Figure 10. DFT calculation of the band structure for 1L-W_{Se}^{S}. **(a)** Energy dispersion plotted along the $\Gamma - M - K - \Gamma$ path. The calculation uses the PBE functional, including spin-orbit coupling. The blue and red arrows indicate the spin of the bands at the K point (spin up and down respectively) and the black arrow shows the energy gap. The Fermi energy is set to zero. **(b)** The exchange energy for the inter-valley negative trion, between the excess electron (grey circle) and the paired electron-hole pair (black and white circle). J_{ex}^{ee} and J_{ex}^{eh} are the electron-electron and electron-hole exchange energies, respectively. σ indicates the paired electron-hole pair. Spin up bands are shown in blue and spin down in red.

Complex	Binding energy (meV)
Negative trion (X^-)	32.2
Positive trion (X^+)	32.0
Biexciton (XX^0)	21.0
Quinton (XX^-)	54.2

Supplementary Table I. **Binding energy of the excitonic complexes compared to the neutral exciton, for free-standing 1L-W_{Se}^{S}.**

Supplementary Note S5. BAND STRUCTURE CALCULATIONS

We perform density functional theory (DFT) to understand the band structure and observed excitonic transitions of 1L-W_{Se}^{S}. Supplementary Figure 10a shows the band structure along the $\Gamma - M - K - \Gamma$ path. This is calculated using the DFT-PBE functional, including spin-orbit coupling with a k-grid of $24 \times 24 \times 1$. The DFT calculation shows that, similar to conventional tungsten-based TMDs (1L-WSe_2 and 1L-WS_2) [17–19], 1L-W_{Se}^{S} is direct bandgap at the K points with spin ordering such that the upper valence band is opposite in spin to the lower spin-split conduction band, with 37 meV spin splitting.

The effective masses obtained from the DFT calculation are used in the Mott-Wannier model and quantum Monte Carlo (QMC) [12, 20] to calculate the expected binding energies of the excitonic charge complexes in free-standing 1L-W_{Se}^{S}, as given in Supplementary Table I. Experimental differences arise from the difference in dielectric environment caused by the ML-hBN encapsulation [21].

The fine structure of the negative trion arises from the exchange interaction between the excess electron and the paired electron-hole, which causes an energy increase for the intervalley relative to intravalley negative trion [22–24]. Supplementary Figure 10b shows the electron-electron (J_{ex}^{ee}) and electron-hole (J_{ex}^{eh}) exchange energies for the intervalley trion.

The magnitude of the exchange energies are [22, 25]:

$$J_{ex}^{ee} \approx g^{ee} V(\mathbf{K}) |\langle u_{\mathbf{K}'c\downarrow} | u_{\mathbf{K}c\downarrow} \rangle|^2$$
$$J_{ex}^{eh} \approx g^{eh} V(\mathbf{K}) |\langle u_{\mathbf{K}'v\downarrow} | u_{\mathbf{K}c\downarrow} \rangle|^2$$

where u are the periodic Bloch wave factors at the K and K' points in the Brillouin zone. The indices $c\downarrow$ and $v\downarrow$ denote the spin down conduction band and spin down valence band, respectively, and correspond to the bands shows in Supplementary Fig. 10b. g^{ee} and g^{eh} are the electron-electron and electron-hole contact pair distribution function (PDF), respectively, which correspond to the probability of the finding the particles in the same position. $V(\mathbf{K})$ is

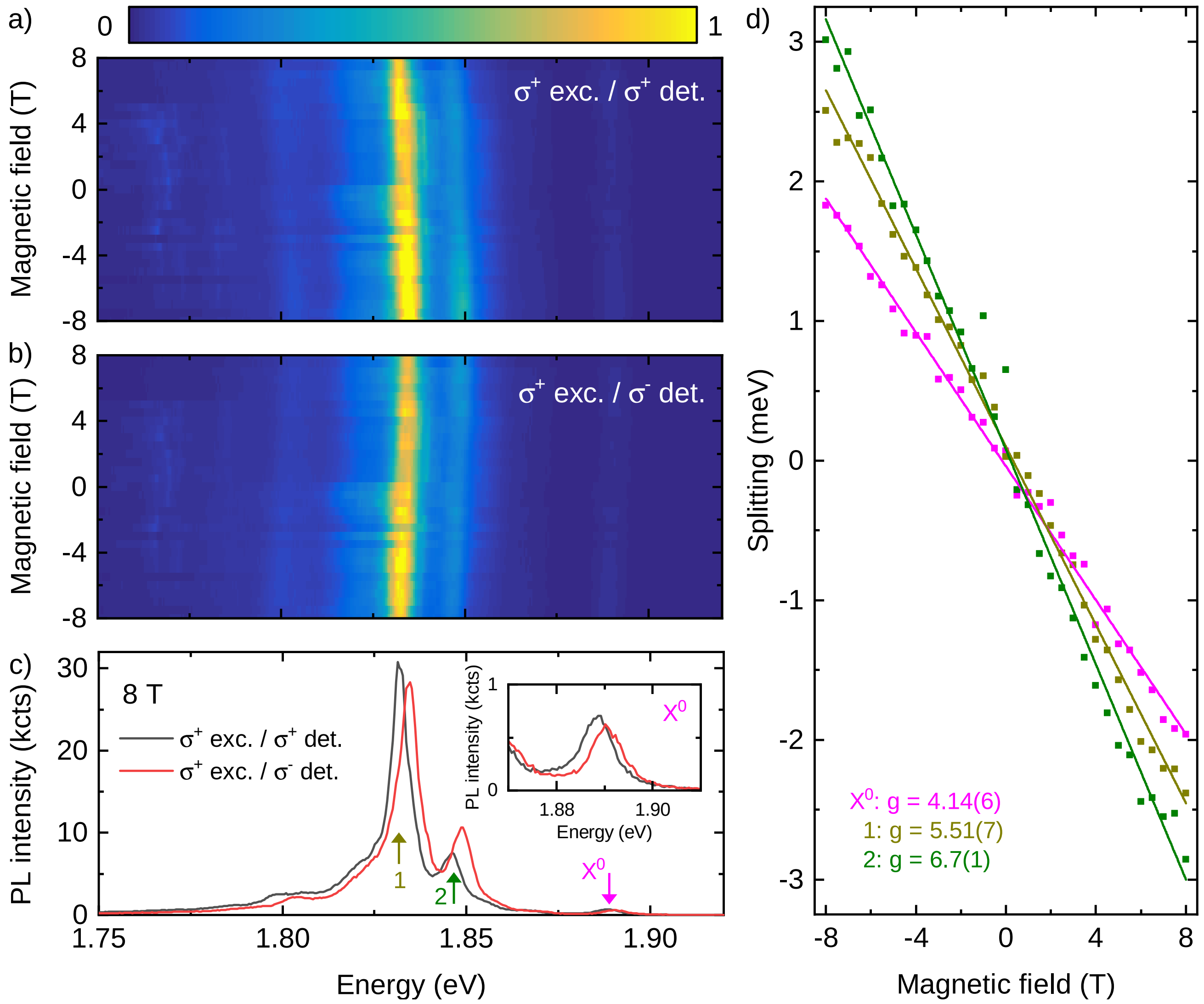

Supplementary Figure 11. Magnetic field dependence of the PL for 1L-W^{S}_{Se}, acquired at 4 K and using 2.33 eV excitation. **(a)** PL spectra as a function of magnetic field, under right-circularly (σ^+) polarised excitation and detection (co-polarised). **(b)** Same as for **a** but with right-circularly (σ^+) polarised excitation and left-circularly (σ^-) polarised detection (cross-polarised). **(c)** PL spectra at 8 T, under σ^+ excitation and with both σ^+ (black curve) and σ^- (red curve) detection. The inset shows only the neutral exciton X^0. **(d)** Energy splitting ΔE as a function of magnetic field B between the σ^+ and σ^- detected PL for the labelled peaks in **c**. The solid lines are linear fittings, with the g factor found from the gradient as $\Delta E = -g\mu_B B$.

the unscreened form of the 2d Coulomb interaction.

We calculate the PDFs for 1L-W^{S}_{Se} by using the quantum Monte Carlo method [20] and find g^{ee} to be much smaller than g^{eh}, which is due to the Coulomb repulsion between two electrons. Hence, we neglect g^{ee} and J^{eh}_{ex} dominates over J^{ee}_{ex}. Using DFT-PBE, we calculate $|\langle u_{\mathbf{K}'v\downarrow}|u_{\mathbf{K}c\downarrow}\rangle|^2 = 0.1$ and therefore we calculate the exchange energy splitting of the negative trion to be $J_{ex} \sim 6$ meV, which agrees well with our observation of an energy difference of 7 meV between X^-_{inter} and X^-_{intra} on device 1 (as discussed in the main text).

Supplementary Note S6. MAGNETIC-FIELD DEPENDENT MEASUREMENTS

We perform a magnetic field dependence of the 1L-W^{S}_{Se} PL emission, with circularly polarised excitation and detection. Supplementary Figs. 11a and b show the PL spectra as a function of magnetic field, under right circularly polarised excitation and for co- and cross-polarised detection, respectively. As we apply the out-of-plane magnetic field, we see the various PL peaks shift in energy. Supplementary Fig. 11c shows the co- and cross-polarised (black

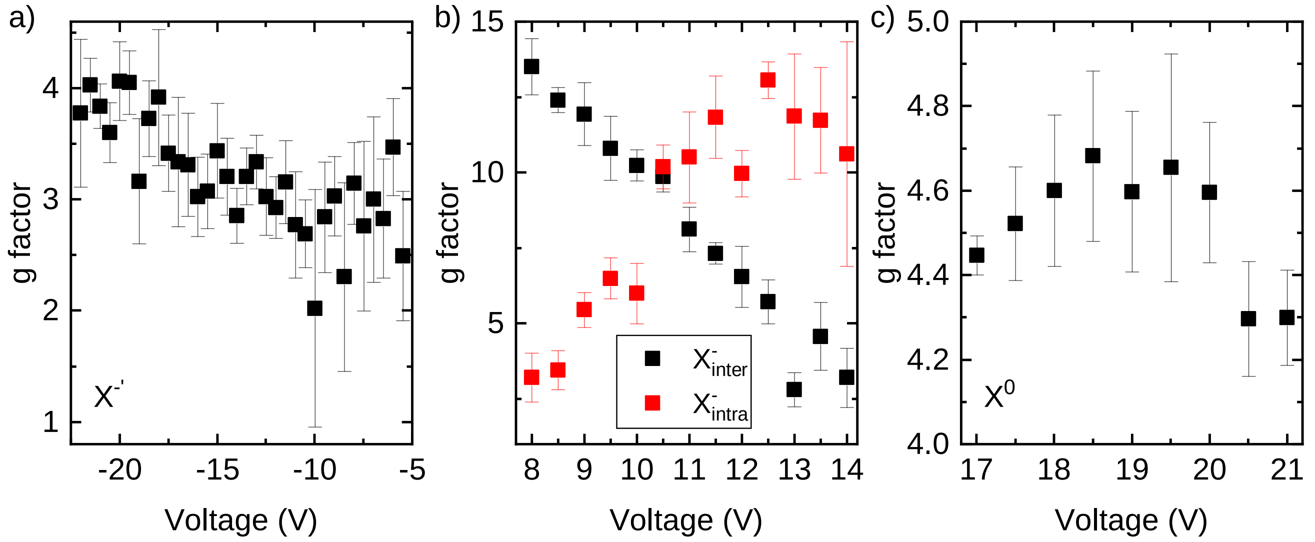

Supplementary Figure 12. Gate voltage dependence of the g factor of the different excitonic species. **(a)** Voltage dependence of the g factor for the $X^{-\prime}$ peak, between -22 to -5 V. **(b)** Voltage dependence of the g factor for the X^-_{inter} (black points) and X^-_{intra} (red points) peaks, between 8 to 14 V. **(c)** Voltage dependence of the g factor for the X^0 peak, between 17 to 21 V. All g factors are extracted from a linear fitting to the energy splitting in RC between σ^+ and σ^- detection polarisation with magnetic field. Error bars are given by the fitting error from the linear fitting.

and red curves, respectively) PL spectra at an applied field of 8 T, with the inset showing the energy splitting for the X^0 peak. The energy splitting ΔE between the opposite circularly polarisations with magnetic field is shown in Supplementary Fig. 11d, for the peaks labelled 1, 2 and X^0. A linear fitting to the energy splitting gives the g factor as $\Delta E = -g\mu_B B$ for each of the peaks: we find a g factor 4.14(6) for the neutral exciton X^0 (as shown in Fig. 4 of the main text), a g factor 5.51(7) for peak 1 and 6.7(1) for peak 2. As discussed in the main text, a g factor $\sim$4 is expected for bright excitons in conventional TMDs. The X^0 g factor from PL agrees with that extracted from RC, 4.5(2), and is as expected for the bright neutral exciton. A higher g factor is also seen for lower energy states in 1L-WSe_2 [26–28] and, similarly to in 1L-WSe_2, these peaks likely arise from various excitonic complexes. Full attribution of the different transitions apparent in PL requires further work.

We find that the g factors, as measured in RC, of the various charged complexes shown in Fig. 4 of the main text have a gate-voltage dependence. Supplementary Figure 12 shows this gate dependence for the $X^{-\prime}$ peak in panel a, X^-_{inter} and X^-_{intra} in panel b (black and red data points, respectively) and X^0 in panel c. At each gate voltage, the g factor is extracted from a linear fitting to the magnetic field dependence of the energy splitting of the peak. We see that for X^0 the g factor remains constant at $\sim$4.5 over the entire range of voltages. The g factor of the $X^{-\prime}$ peak increases as the doping level increases, from $\sim$3 when the peak first appears, towards $\sim$4 V at -20 V. The trion peaks show a larger gate voltage dependence, as discussed in the main text: the inter- and intra-valley trion g factors appear anti-correlated and change from $\sim$3 to $\sim$13. Reported values of the g factors of the negatively charged trions in 1L-WSe_2 vary between similar values [29–32] and this variation is attributed to the doping dependence of the trion recoil effect in ref. [33]. A similar doping dependence of the negatively charged trion in 1L-MoS_2 has also been measured and is attributed to many-body interactions with the Fermi sea of electrons [34].

[1] Nečas, D. & Klapetek, P. Gwyddion: an open-source software for SPM data analysis. *Open Physics* **10**, 181–188 (2012).
[2] Trivedi, D. B. *et al.* Room-temperature synthesis of 2D Janus crystals and their heterostructures. *Advanced Materials* **32**, 2006320 (2020).
[3] Qin, Y. *et al.* Reaching the excitonic limit in 2D Janus monolayers by in situ deterministic growth. *Advanced Materials* **34**, 2106222 (2022).
[4] Krivanek, O. L. *et al.* Atom-by-atom structural and chemical analysis by annular dark-field electron microscopy. *Nature* **464**, 571–574 (2010). URL `https://doi.org/10.1038/nature08879`.
[5] Zhao, W. *et al.* Lattice dynamics in mono-and few-layer sheets of WS_2 and WSe_2. *Nanoscale* **5**, 9677–9683 (2013).
[6] Terrones, H. *et al.* New first order Raman-active modes in few layered transition metal dichalcogenides. *Scientific Reports* **4**, 1–9 (2014).

[7] Petrić, M. M. *et al.* Raman spectrum of Janus transition metal dichalcogenide monolayers WSSe and MoSSe. *Physical Review B* **103**, 035414 (2021).
[8] Duan, X. *et al.* Synthesis of $WS_{2x}Se_{2-2x}$ alloy nanosheets with composition-tunable electronic properties. *Nano Letters* **16**, 264–269 (2016).
[9] Reich, S. *et al.* Resonant Raman scattering in cubic and hexagonal boron nitride. *Physical Review B* **71**, 205201 (2005).
[10] Arenal, R. *et al.* Raman spectroscopy of single-wall boron nitride nanotubes. *Nano Letters* **6**, 1812–1816 (2006).
[11] Ferrari, A. C. *et al.* Raman spectrum of graphene and graphene layers. *Physical Review Letters* **97**, 187401 (2006).
[12] Barbone, M. *et al.* Charge-tuneable biexciton complexes in monolayer WSe_2. *Nature Communications* **9**, 1–6 (2018).
[13] Rodriguez-Pardo Montblanch, A. *Excitons in Two-Dimensional Materials: from Many-Body Physics to Quantum Technologies.* Ph.D. thesis, University of Cambridge (2019).
[14] Kurtsiefer, C., Mayer, S., Zarda, P. & Weinfurter, H. Stable solid-state source of single photons. *Physical Review Letters* **85**, 290 (2000).
[15] He, Y.-M. *et al.* Single quantum emitters in monolayer semiconductors. *Nature Nanotechnology* **10**, 497–502 (2015).
[16] Montblanch, A. R.-P. *et al.* Confinement of long-lived interlayer excitons in WS_2/WSe_2 heterostructures. *Communications Physics* **4**, 119 (2021).
[17] Liu, G.-B., Shan, W.-Y., Yao, Y., Yao, W. & Xiao, D. Three-band tight-binding model for monolayers of group-VIB transition metal dichalcogenides. *Physical Review B* **88**, 085433 (2013).
[18] Kośmider, K., González, J. W. & Fernández-Rossier, J. Large spin splitting in the conduction band of transition metal dichalcogenide monolayers. *Physical Review B* **88**, 245436 (2013).
[19] Kormányos, A. *et al.* k·p theory for two-dimensional transition metal dichalcogenide semiconductors. *2D Materials* **2**, 022001 (2015).
[20] Mostaani, E. *et al.* Diffusion quantum Monte Carlo study of excitonic complexes in two-dimensional transition-metal dichalcogenides. *Physical Review B* **96**, 075431 (2017).
[21] Hsu, W.-T. *et al.* Dielectric impact on exciton binding energy and quasiparticle bandgap in monolayer WS_2 and WSe_2. *2D Materials* **6**, 025028 (2019).
[22] Yu, H., Liu, G.-B., Gong, P., Xu, X. & Yao, W. Dirac cones and Dirac saddle points of bright excitons in monolayer transition metal dichalcogenides. *Nature Communications* **5**, 1–7 (2014).
[23] Plechinger, G. *et al.* Trion fine structure and coupled spin–valley dynamics in monolayer tungsten disulfide. *Nature Communications* **7**, 1–9 (2016).
[24] Courtade, E. *et al.* Charged excitons in monolayer WSe_2: Experiment and theory. *Physical Review B* **96**, 085302 (2017).
[25] Jones, A. M. *et al.* Optical generation of excitonic valley coherence in monolayer WSe_2. *Nature Nanotechnology* **8**, 634–638 (2013).
[26] Koperski, M. *et al.* Orbital, spin and valley contributions to Zeeman splitting of excitonic resonances in $MoSe_2$, WSe_2 and WS_2 monolayers. *2D Materials* **6**, 015001 (2018).
[27] Förste, J. *et al.* Exciton g-factors in monolayer and bilayer WSe_2 from experiment and theory. *Nature Communications* **11**, 1–8 (2020).
[28] He, M. *et al.* Valley phonons and exciton complexes in a monolayer semiconductor. *Nature Communications* **11**, 1–7 (2020).
[29] Koperski, M. *et al.* Single photon emitters in exfoliated WSe_2 structures. *Nature Nanotechnology* **10**, 503–506 (2015).
[30] Wang, G. *et al.* Magneto-optics in transition metal diselenide monolayers. *2D Materials* **2**, 034002 (2015).
[31] Srivastava, A. *et al.* Valley Zeeman effect in elementary optical excitations of monolayer WSe_2. *Nature Physics* **11**, 141–147 (2015).
[32] Koperski, M. *et al.* Optical properties of atomically thin transition metal dichalcogenides: observations and puzzles. *Nanophotonics* **6**, 1289–1308 (2017).
[33] Lyons, T. P. *et al.* The valley Zeeman effect in inter- and intra-valley trions in monolayer WSe_2. *Nature Communications* **10**, 1–8 (2019).
[34] Klein, J. *et al.* Controlling exciton many-body states by the electric-field effect in monolayer MoS_2. *Physical Review Research* **3**, L022009 (2021).
